# Artificial intelligence for evaluation of magnetic resonance imaging-detected extramural vascular invasion in rectal cancer

**DOI:** 10.1371/journal.pdig.0001763

**Published:** 2026-10-01

**Authors:** Haitao Huang, Lili Feng, Min-Er Zhong, Huifen Ye, Zhenhui Li, Su Yao, Yunrui Ye, Yulin Liu, Minning Zhao, Weixiong Xu, Lifen Yan, ChuanMiao Xie, Changhong Liang, Zaiyi Liu, Tong Tong, Yanfen Cui, Xin-Juan Fan, Ke Zhao

**Affiliations:** 1 Department of Radiology, Guangdong Provincial People’s Hospital (Guangdong Academy of Medical Sciences), Southern Medical University, Guangzhou, China; 2 School of Medicine, South China University of Technology, Guangzhou, China; 3 Guangdong Provincial Key Laboratory of Artificial Intelligence in Medical Image Analysis and Application, Guangzhou, China; 4 State Key Laboratory of Oncology in South China, Collaborative Innovation Center for Cancer Medicine, Sun Yat-sen University Cancer Center, Guangzhou, China; 5 Department of Radiology, Sun Yat-sen University Cancer Center, Guangzhou, China; 6 Department of Gastrointestinal Surgery, Department of General Surgery, Guangdong Provincial People’s Hospital (Guangdong Academy of Medical Sciences), Southern Medical University, Guangzhou, China; 7 Department of Radiology, Fudan University Shanghai Cancer Center, Shanghai, China; 8 Department of Oncology, Shanghai Medical College, Fudan University, Shanghai, China; 9 Department of Radiology, The Third Affiliated Hospital of Kunming Medical University, Yunnan Cancer Hospital, Yunnan Cancer Center, Kunming, China; 10 Department of Pathology, Guangdong Provincial People’s Hospital (Guangdong Academy of Medical Sciences), Southern Medical University, Guangzhou, China; 11 Department of Pathology, Heyuan People’s Hospital, Heyuan, China; 12 Heyuan Key Laboratory of Molecular Diagnosis and Disease Prevention and Treatment, Heyuan People’s Hospital, Heyuan, China; 13 Guangdong Cardiovascular Institute, Guangdong Provincial People’s Hospital, Guangdong Academy of Medical Sciences, Guangzhou, China; 14 Department of Radiology, The Sixth Affiliated Hospital, Sun Yat-sen University, Guangzhou, China; 15 Department of Radiology, Zhujiang Hospital, Southern Medical University, Guangzhou, China; 16 Department of Radiology, Shanxi Cancer Hospital, Shanxi Medical University, Taiyuan, China; 17 Department of Pathology, The First Affiliated Hospital of Zhengzhou University, Zhengzhou, Henan, China; 18 Henan Provincial Key Laboratory of Radiation Medicine, The First Affiliated Hospital of Zhengzhou University, Zhengzhou, Henan, China; 19 Tianjian Laboratory of Advanced Biomedical Sciences, Zhengzhou University, Zhengzhou, Henan, China; 20 Chinese Medicine Guangdong Laboratory, Hengqin, China; Tianjin Medical University General Hospital, CHINA

## Abstract

MRI-detected extramural vascular invasion (mrEMVI) is an important prognostic biomarker in rectal cancer, reflecting tumor invasiveness and metastatic potential. To address the subjectivity and inter-observer variability inherent in manual mrEMVI assessment, this study trained and validated an nnUNet-based segmentation model for automated voxel-level localization and visualization of mrEMVI. This multi-center retrospective study included a total of 2,501 rectal cancer patients, comprising 1,830 in the training cohort (with 5-fold cross-validation) and 671 in two independent external test cohorts. The Dice similarity coefficient was used to evaluate segmentation performance; the inter-reader agreement for mrEMVI identification was assessed using Cohen’s kappa (κ). The prognostic value of mrEMVI status identified by the artificial intelligence (AI) model was evaluated using Kaplan-Meier survival analysis and multivariable Cox regression. In internal five-fold cross-validation, the model yielded Dice scores of 0.850, 0.442, and 0.335 for tumor, intravascular tumor signal, and dilated vessel segmentation, respectively. The model demonstrated strong classification performance, with accuracies of 81.5% (95% CI: 76.7%–85.8%) and 84.7% (95% CI: 80.7%–88.2%) in the two external test cohorts, and achieved substantial agreement with senior radiologists (κ = 0.713–0.736). Patients identified as AI-mrEMVI^+^ had significantly lower 3-year disease-free survival (DFS) and 5-year overall survival (OS) rates than AI-mrEMVI^−^ patients (DFS: 62.3% *vs.* 84.9%, HR = 2.67, 95% CI: 1.95–3.66; OS: 68.7% *vs.* 87.1%, HR = 2.64, 95% CI: 1.75–3.97; both *p* < 0.001). This work establishes a scalable, objective framework for mrEMVI assessment based on voxel-level segmentation, inter-observer agreement analysis, and comprehensive prognostic validation, with direct implications for risk stratification and treatment individualization in rectal cancer.

## Author summary

In this study, we aimed to make the evaluation of MRI-detected extramural vascular invasion (mrEMVI) in rectal cancer more objective and reliable. Traditionally, radiologists assess this feature manually on MRI scans, but such evaluations can vary between readers. To address this challenge, we trained a deep learning model based on the nnUNet framework that can automatically identify and outline mrEMVI regions in rectal cancer images. Using MRI data from multiple medical centers, our model learned to recognize these features with high accuracy and achieved substantial agreement with experienced radiologists in mrEMVI identification. We also found that patients identified by the model as AI-mrEMVI^+^ had a significantly higher risk of cancer recurrence and shorter survival times, indicating that our model can provide valuable prognostic information in addition to achieving substantial agreement with experienced radiologists. By offering a consistent and visually interpretable approach to mrEMVI assessment, our work may help doctors make better treatment decisions and move toward more personalized care for people with rectal cancer.

## Introduction

Rectal cancer is a common malignancy with high morbidity and mortality, prompting ongoing investigation into prognostic factors [1]. Although the tumor-node-metastasis (TNM) staging system is widely used, it does not adequately reflect the substantial heterogeneity among patients within the same stage [2,3]. MRI, with its superior soft-tissue contrast, is essential for evaluating the local invasiveness and metastatic potential of rectal tumors [4]. MRI-detected extramural vascular invasion (mrEMVI), a clinically accessible imaging marker, reflects tumor invasiveness and metastatic potential. EMVI refers to the invasion of tumor cells into vessels beyond the muscularis propria [5]. Patients with mrEMVI^+^ rectal cancer have a three- to fourfold higher risk of distant metastasis and local recurrence [6–11]. According to the European Society for Medical Oncology (ESMO) guidelines, mrEMVI^+^ is recognized as a key risk factor in rectal cancer and an indication for neoadjuvant chemoradiotherapy (nCRT) [12,13]. Moreover, mrEMVI assessment has been incorporated into structured clinical reporting, highlighting its clinical relevance [14].

Currently, mrEMVI assessment remains highly subjective and observer-dependent, with reported inter-observer agreement ranging from 0.308 to 0.651 (Cohen’s kappa) [15,16], underscoring the need for more objective and standardized evaluation methods. To address this limitation, radiomics-based approaches have been explored, with a recent meta-analysis reporting pooled sensitivity and specificity of 80% and 89%, respectively, for MRI-based radiomic models in detecting EMVI [17]. However, these methods rely on manual segmentation and handcrafted feature extraction, limiting their reproducibility and clinical scalability. In recent years, deep learning has emerged as a more scalable alternative, demonstrating remarkable advances in medical image analysis [18–21] and significantly improving disease diagnosis, prognosis prediction, and treatment response assessment [22–24]. In particular, segmentation models provide clinicians with precise lesion localization and boundary information, enhancing both classification accuracy and interpretability [25,26]. Numerous studies have demonstrated that artificial intelligence (AI)-based tumor segmentation in MRI matches or even surpasses expert-level performance [27–29]. Among available frameworks, nnUNet enables accurate delineation of organs or pathological lesions in 3D images without requiring manual tuning of network architecture, demonstrating superior robustness and generalizability across diverse medical image segmentation tasks [30–32]. A prior study pioneered the use of deep learning for automated mrEMVI assessment, achieving an area under the receiver operating characteristic curve (AUC) of 0.76 in external validation [24]. However, this approach requires concurrent diffusion-weighted imaging (DWI) and T2-weighted imaging (T2WI) acquisition and provides only binary classification without spatial localization of mrEMVI regions. To the best of our knowledge, no study has applied deep learning to voxel-level anatomical segmentation of mrEMVI, which would simultaneously enable automated status determination, precise lesion localization, and volumetric quantification — capabilities that neither radiomics-based nor classification-based approaches can provide.

In this study, we trained and validated an nnUNet-based segmentation model for the automated identification, localization, and volumetric quantification of mrEMVI on a large-scale annotated multi-center dataset, with the aim of establishing an objective, reproducible framework that bridges automated image analysis and prognostic evaluation in rectal cancer.

## Methods

### 1. Ethics statement

This retrospective multi-center study was approved by the Ethics Committee of Guangdong Provincial People’s Hospital (Approval No. KY2024-673-01). Due to the retrospective design and use of anonymized data, the requirement for written informed consent was waived by all involved committees. This study did not include any participants under 18 years of age.

### 2. Patients

This multi-center retrospective study aimed to train and validate an nnUNet-based segmentation model for automated identification of mrEMVI (AI-mrEMVI model). During the training phase, a total of 1,830 patients from four hospitals were included to form the training cohort. To assess the performance of the model, 671 patients from four hospitals were recruited into two independent external test cohorts. Detailed information on the training and external test cohorts is provided in Text A in S1 Appendix. The specific inclusion and exclusion criteria are outlined in Text B in S1 Appendix. The patient selection and recruitment flowchart is illustrated in Fig A in S1 Appendix. External test cohort 2 comprised patients from two new institutions (GDPH, n = 55; FDCC, n = 134) and a non-overlapping patient cohort from SXCH (n = 184), an institution also represented in the training cohort. At SXCH, training patients underwent direct surgery without nCRT, whereas patients in external test cohort 2 received standard nCRT before surgery, ensuring patient-level independence.

### 3. Follow-up definition and clinical/pathological data collection

This study evaluated two primary outcomes in the two external test cohorts: 3-year disease-free survival (DFS) and 5-year overall survival (OS). Clinical and pathological data were extracted from the medical record system, including age, sex, preoperative carcinoembryonic antigen (CEA) levels, and postoperative pathological tumor regression grade (TRG). The American Joint Committee on Cancer (AJCC) TRG system used in this study comprises four grades, ranging from complete response (TRG 0) to poor response (TRG 3) [33].

### 4. MRI acquisition and imaging biomarker assessment

At the participating hospitals, each patient underwent MRI examinations using 1.5-T or 3.0-T scanners prior to the initiation of treatment. The imaging protocol included T2WI acquired in one or more planes (axial, coronal, and/or sagittal). T2WI acquisition in all three planes was not required; patients were eligible if at least one diagnostic-quality axial, coronal, or sagittal T2WI series was available. Each available plane was processed independently, without imputation or reconstruction of unavailable planes. The MRI acquisition parameters are summarized in Table A in S1 Appendix. The original MRI data were exported as Digital Imaging and Communications in Medicine (DICOM) files from the Picture Archiving and Communication System (PACS).

For patients in the two external test cohorts, baseline imaging variables including mrT stage (mrT1–3 or mrT4), mrN stage (mrN0, mrN1, or mrN2), and tumor location (low, mid, or high) were additionally reassessed by the radiologists participating in this study.

### 5. Determination of the mrEMVI status and manual annotation of tumor and mrEMVI regions

Before image review, all radiologists involved in mrEMVI assessment underwent dedicated training to ensure consistent interpretation of the relevant imaging features. The training focused on the standardized criteria proposed by Smith et al. [6], which define mrEMVI as a continuous extension of tumor signal from the primary lesion along a vascular pathway, accompanied by vascular contour expansion or irregularity. Specifically, training comprised a systematic review of key imaging features, case-based teaching sessions using representative and challenging examples independent of the study dataset, and structured consensus discussions. All subsequent mrEMVI assessments were completed within one month after training.

For all the patients in the training and external test cohorts, baseline mrEMVI status was then independently assessed by two abdominal radiologists, each with 5 years of experience, who reviewed all imaging data while being blinded to clinical, pathological, and follow-up information. Cases with discrepant assessments between the two junior readers, as well as those deemed difficult to evaluate, were referred to two senior abdominal radiologists, each with over 20 years of experience, who jointly reviewed these cases and reached a final consensus on mrEMVI status through discussion (Fig B in S1 Appendix).

After the final consensus mrEMVI status had been established, all MRI examinations from all participating centers (1,830 patients in the training cohort and 671 patients in the external test cohorts) were centrally annotated by a single designated radiologist with 5 years of experience in abdominal lesion diagnosis, under the supervision of a radiologist with 20 years of experience in the same field, using ITK-SNAP software (version 4.1.0). This centralized annotation strategy, rather than distributed local annotation at each center, was adopted to ensure consistency across all participating centers. For mrEMVI^+^ patients, both the primary tumor and mrEMVI lesions were segmented; for mrEMVI^−^ patients, only the primary tumor was annotated. Manual annotation was performed slice-by-slice on all available axial, coronal, and/or sagittal T2WI, with other sequences available for reference (Fig 1A). Consistent with the Smith et al. [6] criteria used during training, the annotated mrEMVI regions included dilated vascular structures and intravascular tumor signals.

**Fig 1:**
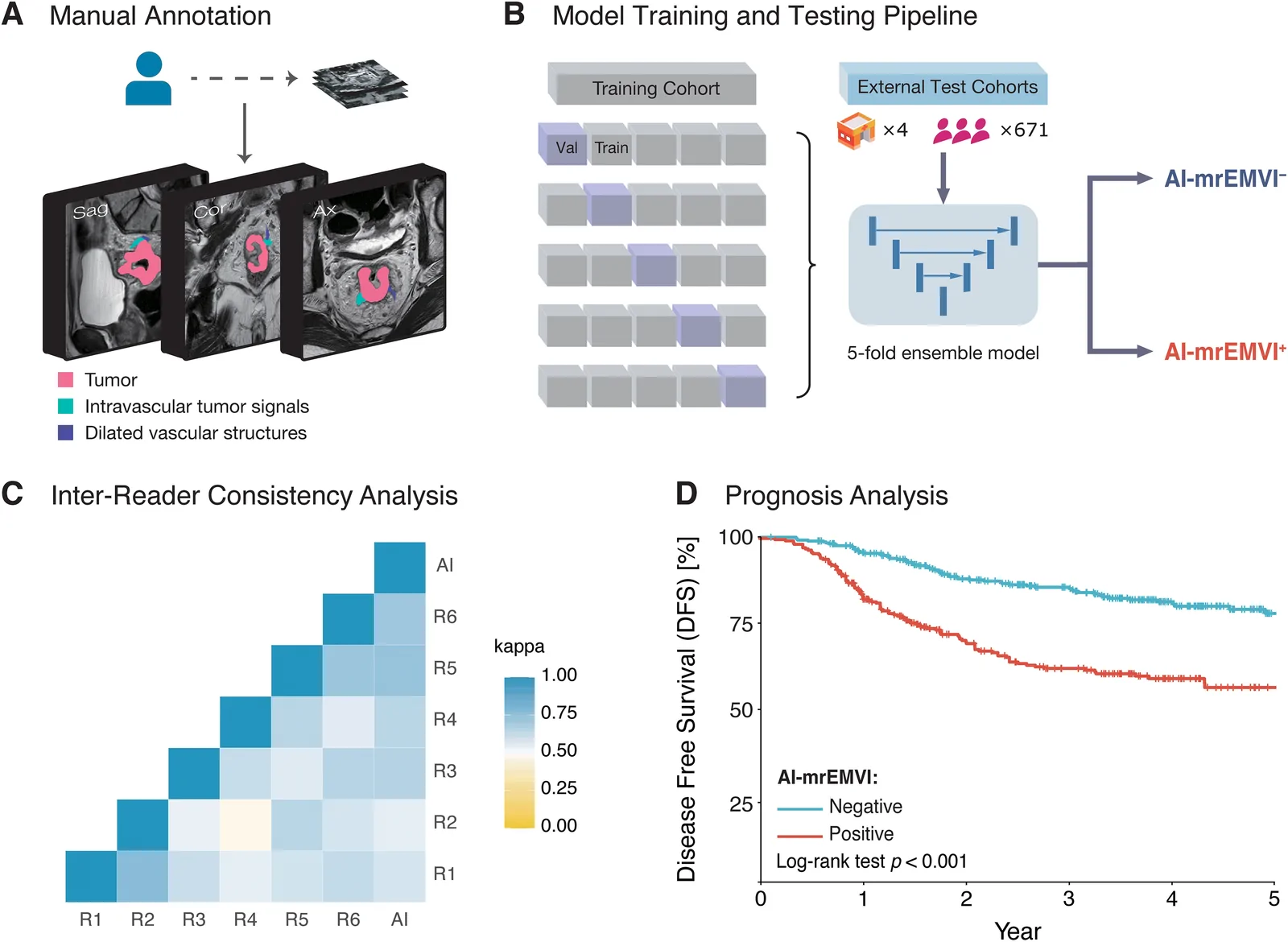
Workflow of the AI-mrEMVI segmentation model from manual annotation to clinical validation. **(A)** Slice-by-slice manual annotation of multiplanar T2-weighted MR images performed by a radiologist, with annotated regions including the primary tumor (pink), intravascular tumor signals (green), and dilated vascular structures (purple). **(B)** Model Training and Testing Pipeline. The segmentation training dataset was divided into training and validation subsets at the plane-specific volume level using five-fold cross-validation. The trained five-fold ensemble model was then applied to two external test cohorts recruited from 4 independent centers (n = 671). **(C)** Pairwise inter-reader consistency analysis presented as a Kappa heatmap across six radiologists of varying experience levels and the AI model. **(D)** Kaplan-Meier curves illustrating the prognostic value of AI-mrEMVI status for disease-free survival. AI, artificial intelligence; MRI, magnetic resonance imaging; EMVI, extramural vascular invasion; R, reader; Val, validation fold.

### 6. Training and configuration of the AI-mrEMVI segmentation model

For segmentation model training, all 499 mrEMVI^+^ patients and 247 selected mrEMVI^−^ patients from the 1,830-patient training cohort were included, yielding a segmentation training dataset of 746 patients with an approximate positive-to-negative ratio of 2:1. These patients contributed 1,289 manually annotated plane-specific T2WI volumes, which served as the ground-truth dataset (Fig C in S1 Appendix). Each plane-specific T2WI volume was treated as a separate training sample and was annotated according to the mrEMVI features visible in that plane, irrespective of the patient-level mrEMVI status. Consequently, different imaging planes from the same patient could have different plane-specific mrEMVI annotations.

Prior to model training, all MR images underwent standardized preprocessing, including non-zero cropping, resampling to an in-plane resolution of 0.3516 mm with a slice thickness of 3.3 mm, and z-score normalization. To enhance model generalizability, data augmentation techniques such as random rotation, scaling, elastic deformation, gamma correction, mirroring, Gaussian noise, and Gaussian blur were applied with a probability of 0.5. Detailed training procedures are provided in Text C in S1 Appendix. All preprocessing parameters, network architecture, loss function, and training strategy were automatically determined by the nnUNet v2 framework based on the dataset fingerprint, without manual tuning or modifications [30].

The standard nnUNet v2 framework was applied to achieve automatic segmentation of the mrEMVI regions [30]. Specifically, the model was trained on multiplanar T2WI and their corresponding manual annotations using the 3D full-resolution UNet architecture, which fully preserves the spatial resolution of the input images. Consistent with this plane-specific annotation and training strategy, five-fold cross-validation was performed at the plane-specific volume level. Across folds 0–4, the training subsets included 662/369, 672/359, 671/360, 665/366, and 670/362 mrEMVI^+^/mrEMVI^−^ plane-specific T2WI volumes, respectively (Fig C in S1 Appendix). Based on the cross-validation results, an ensemble strategy was applied to combine predictions from all five trained models (Fig 1B).

The trained ensemble model was applied to segment MR images from patients in the external test cohorts. During inference, the input images underwent the same preprocessing steps, and ensemble predictions from the five folds were combined by averaging the softmax probabilities. The model generated segmentation masks with four labels: background (Label 0), primary tumor (Label 1), and mrEMVI regions comprising intravascular tumor signals (Label 2) and dilated vascular structures (Label 3). A patient was classified as AI-mrEMVI^+^ if at least one voxel labeled as mrEMVI was detected in any available plane-specific output mask. The complete inference workflow is provided in Text D in S1 Appendix.

To justify the selection of nnUNet v2, comparative experiments were conducted with three established segmentation architectures: SegResNet [34], Swin UNETR [35], and UNETR++ [36]. All models were trained on the same dataset, with each architecture configured according to its officially recommended settings and default preprocessing pipeline to ensure evaluation under optimal conditions. Segmentation performance was evaluated using out-of-fold predictions from internal five-fold cross-validation, whereas classification performance was evaluated in the combined external test cohorts.

### 7. Consistency analysis

To assess the diagnostic consistency between the AI-mrEMVI model and radiologists of different experience levels, a consecutive series of 100 patients was selected from the external test cohorts. The mrEMVI status of each case was independently assessed by six radiologists. The junior radiologist group consisted of two readers with 3–5 years of experience (H.H., Y.Y., denoted as Readers 1 and 2). The mid-level group comprised two readers with 5–10 years of experience (H.Y., Y.L., Readers 3 and 4), while the senior group included two radiologists with over 10 years of experience (L.Y., W.X., Readers 5 and 6). To ensure objectivity, all radiologists were blinded to clinical, pathological, and follow-up information throughout the assessment (Fig 1C).

Additionally, to evaluate the inter-observer reproducibility of manual spatial annotations, a mid-level radiologist with 6 years of experience, who was independent of all readers involved in mrEMVI status adjudication and inter-reader consistency analysis and had participated in all pre-study mrEMVI training sessions, independently annotated the tumor and mrEMVI regions in the same cohort of 100 patients, without access to the primary annotator’s delineations. Dice similarity coefficients between the primary and secondary annotations were then calculated for each mrEMVI region to quantify spatial agreement.

### 8. Statistical analysis

All performance metrics were reported with 95% confidence intervals (CIs), calculated using the Wilson score interval for proportions, DeLong’s method for AUC, the standard error of the mean for Dice scores, and a 1,000-iteration bootstrap resampling procedure for optimism-corrected concordance indices (C-indices).

Inter-reader agreement for mrEMVI was calculated using Cohen’s kappa (κ). The classification performance of the model was evaluated using metrics including accuracy, sensitivity, specificity, positive predictive value (PPV), negative predictive value (NPV), and AUC. Kaplan-Meier curves were plotted to compare the 3-year DFS and 5-year OS rates in each external test cohort. Variables with *p* < 0.05 in univariable analysis were included in multivariable Cox regression using forward stepwise selection to identify independent prognostic factors. Hazard ratios (HRs) with 95% CIs were estimated. The prognostic value of AI-derived mrEMVI was evaluated by comparing three multivariable Cox models, with model performance assessed using the C-index and bootstrap validation. To determine the optimal cutoff values of AI-derived mrEMVI lesion volume for DFS and OS, maximally selected rank statistics were applied using the *surv_cutpoint* function in the R package *survminer*. To evaluate the reliability of AI-derived mrEMVI lesion volume measurements, Spearman’s rank correlation analysis and Bland-Altman analysis were performed between AI-derived and manually annotated volumes in true-positive cases. Statistical significance was set at *p* < 0.05. All analyses were performed using Python (version 3.9) and R (version 4.3.1).

## Results

### 1. Patients

In the overall training cohort, a total of 1,830 patients were included in the final analysis out of 2,150 patients retrospectively recruited from four centers, with 320 patients excluded because they did not meet the inclusion criteria. The prevalence of mrEMVI positivity in the training cohort was 27.3% (499/1,830).

External test cohort 1 comprised 298 patients (211 males, 87 females), including 81 mrEMVI^+^ and 217 mrEMVI^−^ patients. External test cohort 2 comprised 373 patients (239 males, 134 females), including 155 mrEMVI^+^ and 218 mrEMVI^−^ patients. The clinical and radiological characteristics of the two external test cohorts are summarized in Table 1.

**Table 1:** The distributions of demographic and tumor characteristics of rectal cancer patients in two external test cohorts.

	External Test Cohort 1 NO. (%)	External Test Cohort 2 NO. (%)
Age (years)		
< 60 years	181 (60.7)	230 (61.7)
≥ 60 years	117 (39.3)	143 (38.3)
Sex		
Male	211 (70.8)	239 (64.1)
Female	87 (29.2)	134 (35.9)
CEA level^a^		
Normal	178 (59.7)	281 (75.3)
Abnormal	120 (40.3)	90 (24.1)
NA	0 (0)	2 (0.5)
Location		
Low	159 (53.4)	174 (46.6)
Middle	130 (43.6)	181 (48.5)
High	9 (3.0)	16 (4.3)
NA	0 (0)	2 (0.5)
mrT stage		
T1–T2	6 (2.0)	5 (1.3)
T3	245 (82.2)	296 (79.4)
T4	47 (15.8)	72 (19.3)
mrN stage		
N0	80 (26.8)	63 (16.9)
N1	107 (35.9)	132 (35.4)
N2	111 (37.2)	178 (47.7)
mrEMVI status		
Negative	217 (72.8)	218 (58.4)
Positive	81 (27.2)	155 (41.6)
TRG		
0	86 (28.9)	49 (13.1)
1	96 (32.2)	77 (20.6)
2	106 (35.6)	119 (31.9)
3	10 (3.4)	61 (16.3)
NA	0 (0)	67 (18.0)

CEA, carcinoembryonic antigen; MR, magnetic resonance; EMVI, extramural vascular invasion; TRG, tumor regression grade; NA, not applicable.

Unless otherwise indicated, data are numbers of patients, and data in parentheses are percentages.

^a^CEA level was categorized as normal (≤5 ng/mL) or abnormal (>5 ng/mL) based on standard clinical thresholds.

### 2. Segmentation performance of the AI-mrEMVI model

Representative segmentation results are shown in Fig 2. In internal five-fold cross-validation, the Dice scores were 0.850 (95% CI: 0.842–0.858) for the tumor region, 0.442 (95% CI: 0.414–0.469) for the intravascular tumor signal, and 0.335 (95% CI: 0.316–0.354) for the dilated vessel region. In the combined external test cohorts, the corresponding Dice scores were 0.748 (95% CI: 0.730–0.766), 0.340 (95% CI: 0.311–0.354), and 0.256 (95% CI: 0.220–0.283), respectively. Using the pooled out-of-fold predictions from internal five-fold cross-validation, plane-specific volumes were stratified by the median AI-mrEMVI volume (711.91 mm^3^). The Dice scores for the intravascular tumor signal and dilated vessel region were 0.591 (95% CI: 0.561–0.621) and 0.353 (95% CI: 0.328–0.378) in the larger-volume group, compared with 0.304 (95% CI: 0.257–0.351) and 0.320 (95% CI: 0.292–0.347) in the smaller-volume group, respectively. Fig D in S1 Appendix shows comparisons between manual annotations and model-predicted mrEMVI regions across a range of Dice scores in the external test cohorts, including AI-mrEMVI^+^ cases that were correctly classified despite relatively low Dice scores. Fig E in S1 Appendix presents representative examples of typical, ambiguous, and misclassified cases.

**Fig 2:**
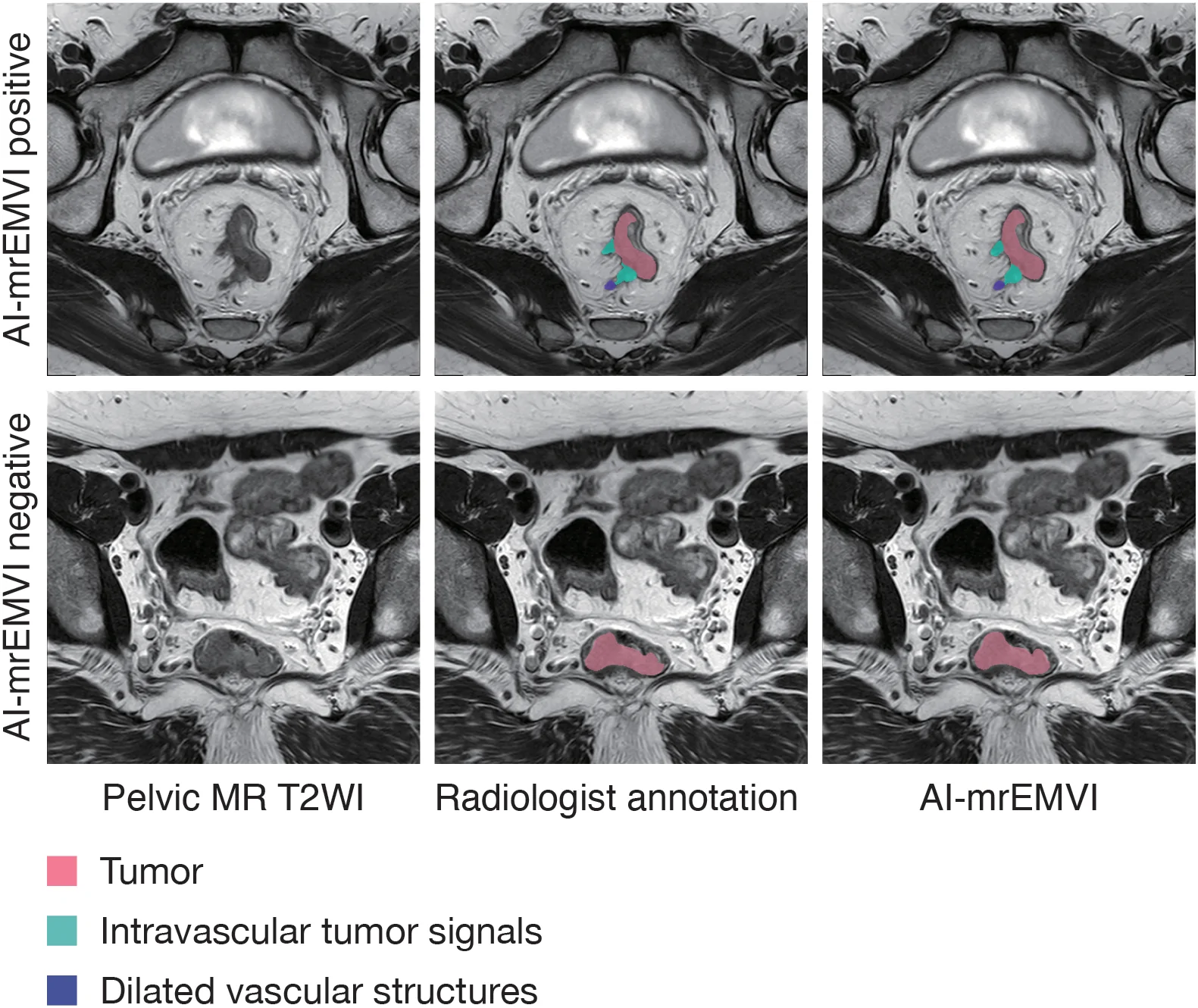
Segmentation results of the AI-mrEMVI model for AI-mrEMVI^−^ and AI-mrEMVI^+^ cases. From left to right, the images show the T2WI, manual annotations by radiologists, and segmentation results generated by the AI-mrEMVI model. Color overlays: Red indicates the primary tumor tissue; green indicates intravascular tumor signals; purple indicates dilated vascular structures. AI, artificial intelligence; mr, magnetic resonance; EMVI, extramural vascular invasion; T2WI, T2-weighted image.

Among the four evaluated architectures, nnUNet v2 achieved the best segmentation performance across all regions in internal five-fold cross-validation (Table B in S1 Appendix).

### 3. Classification performance of the AI-mrEMVI model

Model predictions in the two external test cohorts were compared with the ground truth established by the senior radiologist panel, and the resulting confusion matrices are shown in Fig 3A–B. The model achieved classification accuracies of 81.5% (95% CI: 76.7%–85.8%) in external test cohort 1 and 84.7% (95% CI: 80.7%–88.2%) in external test cohort 2. Across the combined external test cohorts, the model achieved an overall sensitivity of 89.0% (95% CI: 84.3%–92.4%), specificity of 80.2% (95% CI: 76.2%–83.7%), PPV of 70.9% (95% CI: 65.5%–75.8%), and NPV of 93.1% (95% CI: 90.0%–95.4%) (Fig 3C). Additionally, based on the binary classification results, the AUCs were 0.819 (95% CI: 0.770–0.868) in external test cohort 1 and 0.858 (95% CI: 0.824–0.892) in external test cohort 2 (Fig 3C). Site-stratified analysis of external test cohort 2 showed comparable model performance between SXCH and the institutionally independent sites (GDPH and FDCC). The SXCH patients included in the training and external test cohorts were non-overlapping, suggesting that institutional-level overlap had minimal influence on external test performance (Fig F in S1 Appendix).

**Fig 3:**
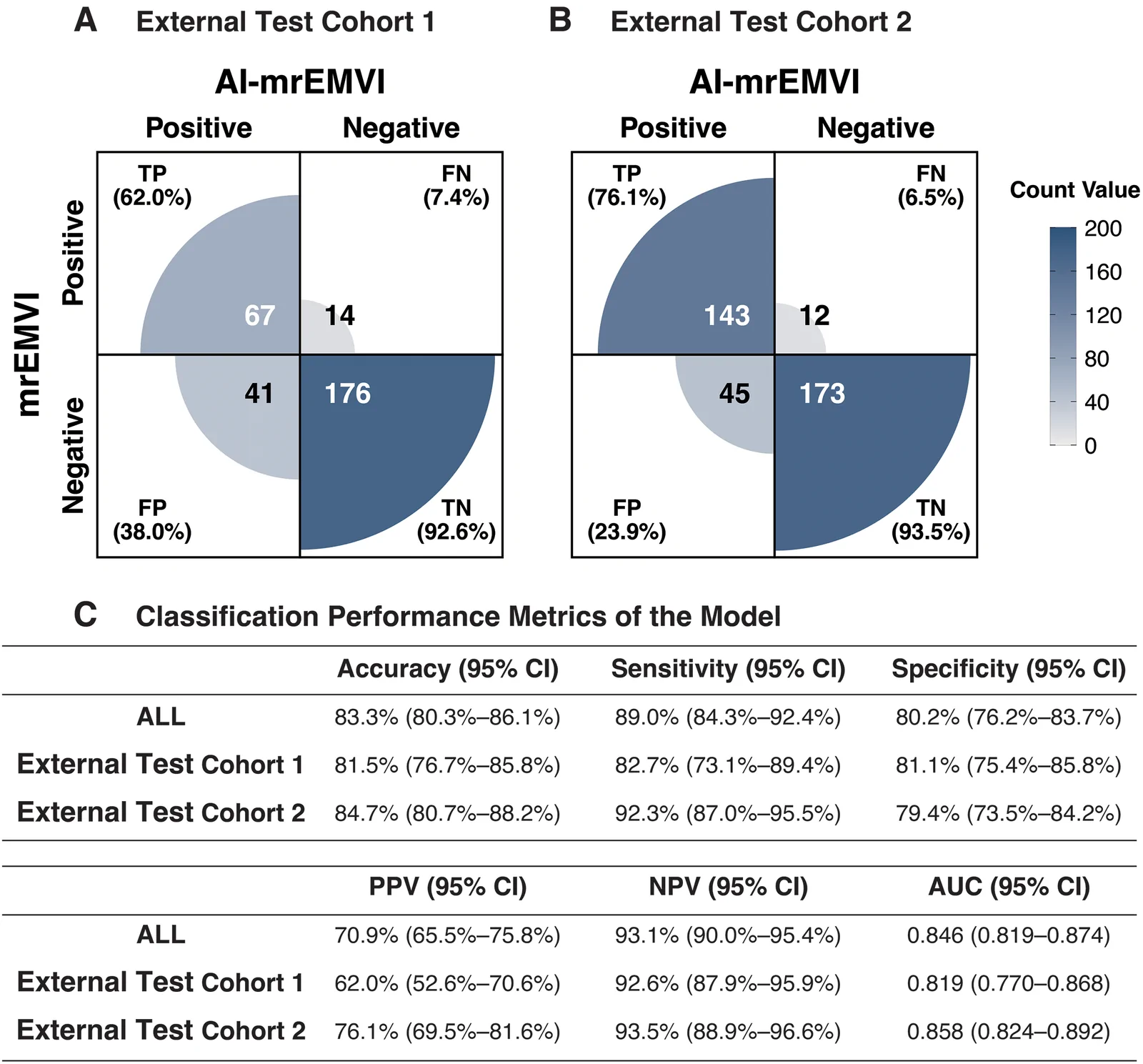
Confusion matrices and performance metrics of the AI-mrEMVI model. **(A)** Confusion matrix for external test cohort 1. **(B)** Confusion matrix for external test cohort 2. **(C)** Classification performance metrics of the model. AI, artificial intelligence; mr, magnetic resonance; EMVI, extramural vascular invasion; T2WI, T2-weighted image; PPV, Positive Predictive Value; NPV, Negative Predictive Value; AUC, Area Under the ROC Curve.

Among the four evaluated architectures, nnUNet v2 achieved the highest overall classification accuracy of 83.3% (95% CI: 80.3%–86.1%) in the combined external test cohorts, outperforming SegResNet (71.2%, 95% CI: 67.7%–74.5%), Swin UNETR (68.9%, 95% CI: 65.2%–72.2%), and UNETR++ (51.6%, 95% CI: 47.8%–55.3%). UNETR++ showed a high sensitivity of 93.2% (95% CI: 89.3%–95.8%) but a low specificity of 29.0% (95% CI: 24.9%–33.4%), indicating a tendency to overpredict mrEMVI^+^ cases (Table C in S1 Appendix).

### 4. Consistency analysis

The AI-mrEMVI model demonstrated high agreement with senior radiologists in identifying mrEMVI, with Cohen’s kappa values of 0.713 (standard error [SE]: 0.045, 95% CI: 0.625–0.802) and 0.736 (SE: 0.044, 95% CI: 0.650–0.823) (Fig 4). Moreover, the AI-mrEMVI model showed substantial agreement with mid-level radiologists, with Cohen’s kappa values of 0.662 (SE: 0.047, 95% CI: 0.569–0.754) and 0.649 (SE: 0.048, 95% CI: 0.556–0.743). No statistically significant difference was observed between AI–senior radiologist agreement and senior–mid-level radiologist agreement (Z = 1.787, *p* = 0.074).

**Fig 4:**
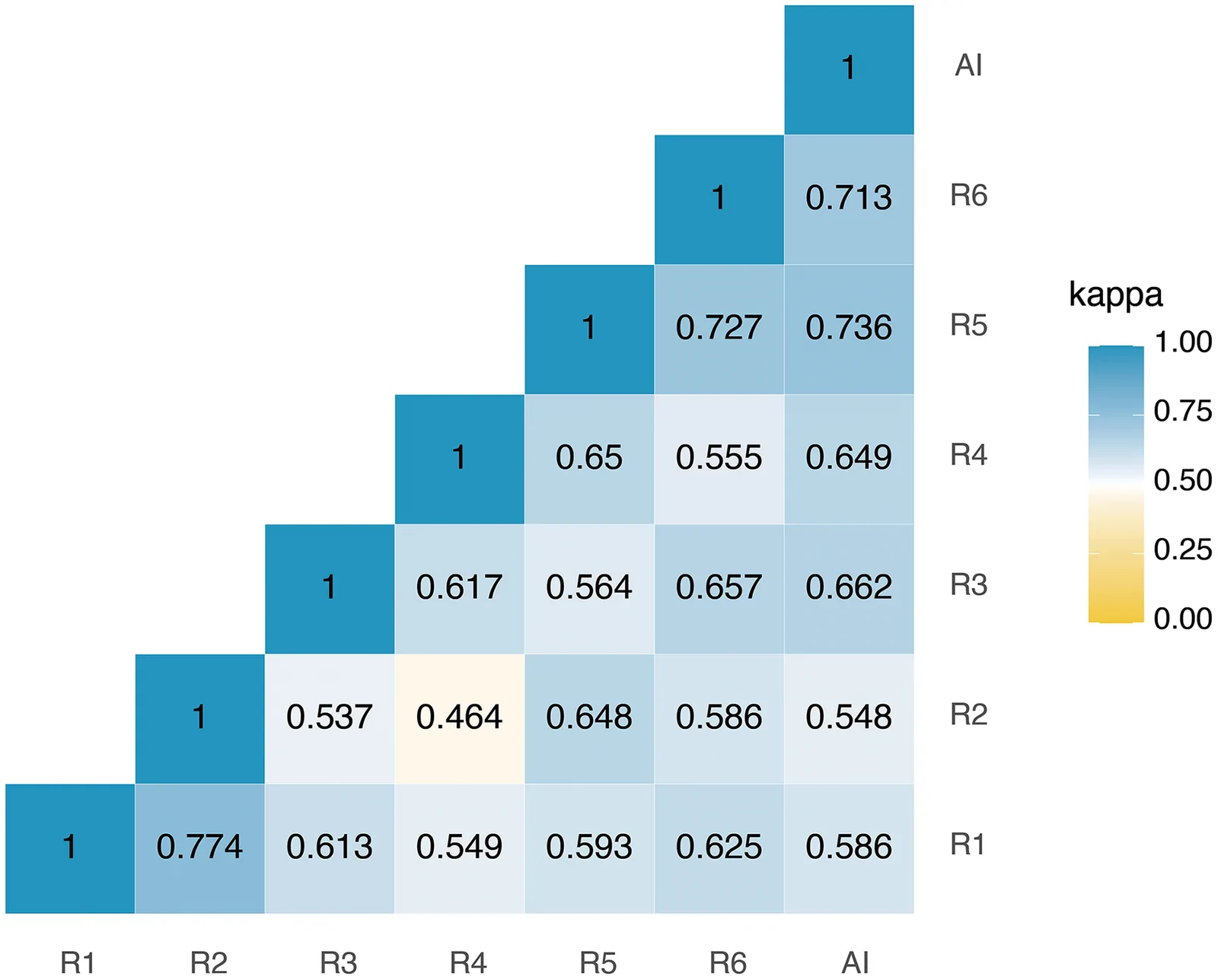
Heatmap of inter-reader agreement between the AI-mrEMVI model and radiologists in mrEMVI assessment. The color intensity in the heatmap represents the pairwise Cohen’s kappa values between each pair of readers. R1 and R2 denote junior radiologists (H.H. and Y.Y.); R3 and R4, mid-level radiologists (H.Y. and Y.L.); and R5 and R6, senior radiologists (L.Y. and W.X.). R, reader. AI, artificial intelligence; mr, magnetic resonance; EMVI, extramural vascular invasion.

The primary and secondary annotators achieved Dice coefficients of 0.653 (95% CI: 0.640–0.667) for the intravascular tumor signal region and 0.321 (95% CI: 0.309–0.335) for the dilated vessel region. For mrEMVI classification, Cohen’s kappa between the two readers was 0.694 (SE: 0.046, 95% CI: 0.603–0.784).

### 5. Prognostic value of the AI-mrEMVI model

The median follow-up duration was 3.75 years (interquartile range [IQR], 2.48–4.59). In both external test cohorts, AI-mrEMVI^+^ patients had poorer DFS and OS than AI-mrEMVI^−^ patients (Fig 5A–D). Specifically, the 3-year DFS rates were 62.3% and 84.9%, respectively (HR = 2.67, 95% CI: 1.95–3.66, *p* < 0.001), and the 5-year OS rates were 68.7% and 87.1%, respectively (HR = 2.64, 95% CI: 1.75–3.97, *p* < 0.001) (Fig G in S1 Appendix).

**Fig 5:**
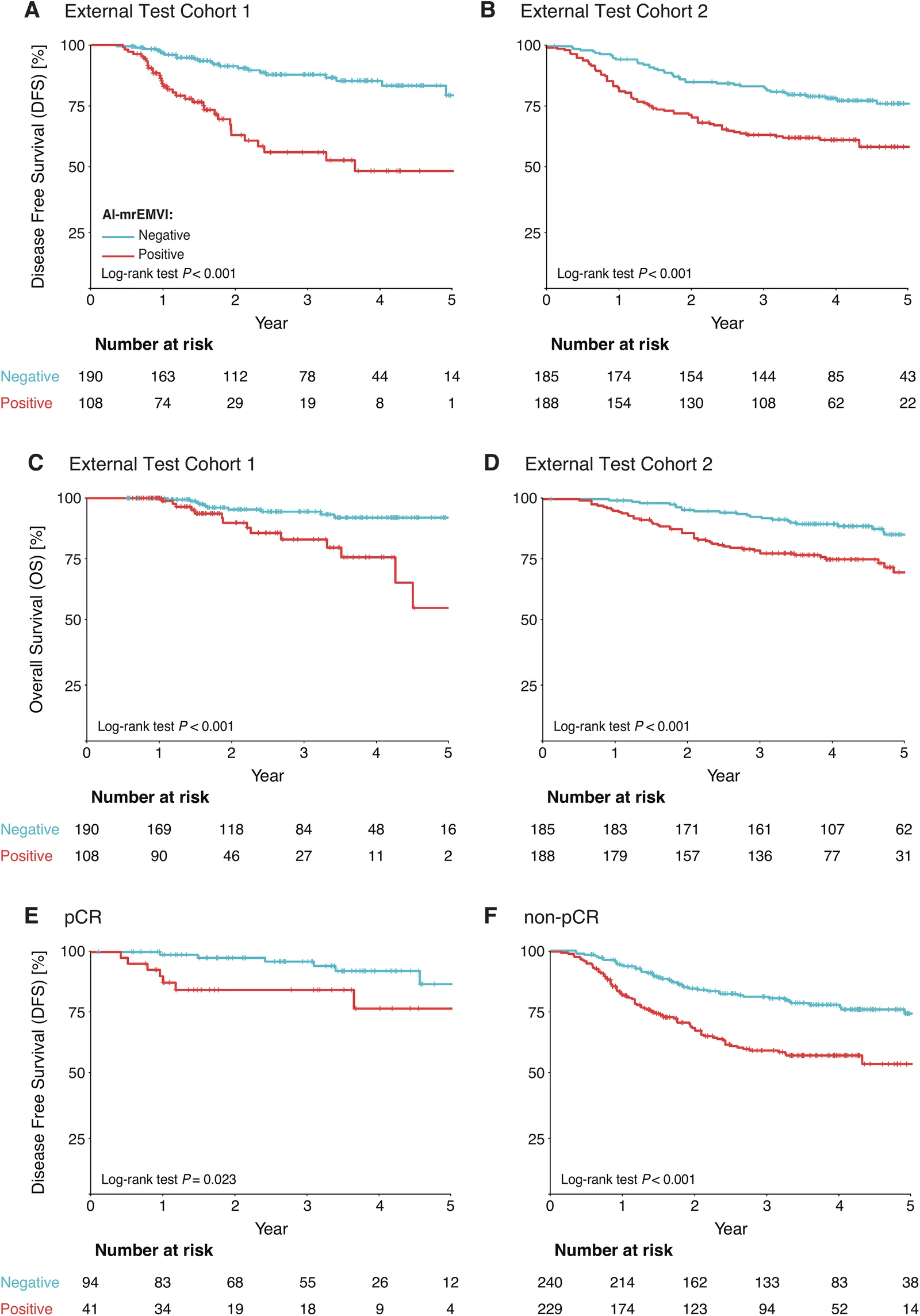
The prognostic value of AI-mrEMVI status. Kaplan-Meier survival curves showing DFS in external test cohort 1 **(A)** and external test cohort 2 **(B)**. Kaplan–Meier survival curves for OS in external test cohort 1 **(C)** and external test cohort 2 **(D)**. Kaplan–Meier survival curves for patients who achieved a pCR **(E)** and those who did not (non-pCR) **(F)** after receiving nCRT. AI, artificial intelligence; mr, magnetic resonance; EMVI, extramural vascular invasion; DFS, disease-free survival; OS, overall survival; pCR, pathological complete response; nCRT, neoadjuvant chemoradiotherapy.

To evaluate the incremental prognostic value of AI-mrEMVI, three multivariable Cox regression models were established: (1) a baseline clinical model (including mrT, mrN, CEA level, and tumor location); (2) the baseline clinical model plus expert radiologist consensus-determined mrEMVI status; and (3) the baseline model plus AI-mrEMVI status. After bootstrap correction, the C-indices for DFS in these three models were 0.616 (95% CI: 0.575–0.656), 0.658 (95% CI: 0.620–0.700), and 0.677 (95% CI: 0.638–0.719), respectively; the corresponding C-indices for OS were 0.680 (95% CI: 0.626–0.735), 0.706 (95% CI: 0.654–0.761), and 0.711 (95% CI: 0.661–0.769), respectively (Fig H in S1 Appendix). Compared with the model containing clinical variables alone, the inclusion of either radiological expert consensus mrEMVI status or AI-mrEMVI significantly improved the prognostic discrimination. The differences between the models incorporating AI-mrEMVI and radiological expert consensus mrEMVI status were not statistically significant for either endpoint (Table D in S1 Appendix).

To further examine the prognostic implications of discordant assessments between AI and radiologist-established ground truth (GT), Kaplan-Meier analyses were performed comparing two discordant subgroups: false-positive cases (AI-mrEMVI^+^/GT-mrEMVI^−^, n = 86) and false-negative cases (AI-mrEMVI^−^/GT-mrEMVI^+^, n = 26). No statistically significant differences were observed between the two groups in DFS or OS (DFS: *p* = 0.43; OS: *p* = 0.31) (Fig I in S1 Appendix). Representative examples of AI misclassification are illustrated in Fig E in S1 Appendix.

Univariable analysis in external test cohort 1 revealed that pre-treatment CEA levels, mrN stage, and AI-mrEMVI status were significantly associated with DFS. In external test cohort 2, univariable analysis demonstrated significant associations of tumor location, mrN stage, and AI-mrEMVI status with DFS. In external test cohort 1, multivariable Cox regression analysis for DFS revealed that AI-mrEMVI^+^ (adjusted hazard ratio [AHR] = 3.78, 95% CI: 2.18–6.58; *p* < 0.001) and abnormal CEA levels (AHR = 1.83, 95% CI: 1.07–3.15; *p* = 0.027) were independently predictive of increased recurrence risk (Table 2). In external test cohort 2, multivariable analysis for DFS demonstrated that AI-mrEMVI^+^ (AHR = 2.33, 95% CI: 1.55–3.49; *p* < 0.001) and mrN2 stage (AHR = 1.94, 95% CI: 1.05–3.58; *p* = 0.034) were independently associated with increased recurrence risk, whereas higher tumor location was independently associated with reduced recurrence risk (AHR = 0.47, 95% CI: 0.32–0.70; *p* < 0.001) (Table 2). Results of the univariable and multivariable OS analyses are presented in Table E in S1 Appendix. Schoenfeld residual tests confirmed that the proportional hazards assumption was satisfied in all multivariable Cox models (Table F in S1 Appendix).

**Table 2:** Univariable and multivariable analyses of DFS.

	Disease-free survival (External Test Cohort 1)				Disease-free survival (External Test Cohort 2)			
	Univariable analysis		Multivariable analysis		Univariable analysis		Multivariable analysis	
	HR (95% CI)	*p*	AHR (95% CI)	*p*	HR (95% CI)	*p*	AHR (95% CI)	*p*
Age	1.01 (0.98–1.03)	0.575			1.00 (0.99–1.02)	0.726		
Sex								
Female	Ref				Ref			
Male	1.29 (0.70–2.36)	0.415			1.04 (0.64–1.68)	0.879		
CEA level^a^								
Normal	Ref		Ref		Ref			
Abnormal	2.29 (1.35–3.90)	0.002	1.83 (1.07–3.15)	0.027	1.14 (0.75–1.73)	0.551		
Location								
Low	Ref				Ref		Ref	
Middle & High	1.09 (0.64–1.84)	0.870			0.63 (0.43–0.91)	0.015	0.47 (0.32–0.70)	< 0.001
mrT stage								
T1–T3	Ref				Ref			
T4	1.24 (0.64–2.39)	0.526			1.81 (1.20–2.73)	0.005		
mrN stage								
N0	Ref				Ref		Ref	
N1	2.12 (0.99–4.55)	0.053			1.42 (0.75–2.68)	0.282	1.28 (0.67–2.45)	0.452
N2	2.22 (1.02–4.84)	0.044			2.13 (1.17–3.87)	0.013	1.94 (1.05–3.58)	0.034
AI-mrEMVI status								
Negative	Ref		Ref		Ref		Ref	
Positive	4.22 (2.45–7.29)	< 0.001	3.78 (2.18–6.58)	< 0.001	2.13 (1.45–3.13)	< 0.001	2.33 (1.55–3.49)	< 0.001

HR, hazard ratio; AHR, adjusted hazard ratio; CI, confidence interval; mr, magnetic resonance; CEA, carcinoembryonic antigen; EMVI, extramural vascular invasion; AI, artificial intelligence.

^a^CEA level was categorized as normal (≤5 ng/mL) or abnormal (>5 ng/mL) based on standard clinical thresholds.

The AI-mrEMVI volume was calculated for each patient to evaluate its prognostic value. To avoid optimism bias, the optimal cutoff values for DFS and OS were derived using maximally selected rank statistics applied exclusively to external test cohort 1 (DFS: 2,329.16 mm^3^; OS: 2,729.58 mm^3^) and subsequently applied unchanged to external test cohort 2 as an independent validation dataset. Based on these thresholds, patients were stratified into high- and low-volume groups. Kaplan-Meier analysis in external test cohort 2 confirmed significantly worse DFS and OS in the high-volume group compared with the low-volume group (DFS: *p* < 0.001; OS: *p* = 0.005) (Fig J in S1 Appendix). To further evaluate the reliability of AI-derived volume measurements, a correlation analysis was performed between AI-derived and manually annotated mrEMVI volumes in true-positive cases. Among true-positive cases with available manual annotations, AI-derived volume was significantly correlated with manually annotated volume (Spearman’s ρ = 0.793, *p* < 0.001; Bland-Altman mean difference: 619.51 mm^3^, 95% limits of agreement [LoA]: −1393.74 to 2632.75 mm^3^) (Fig K in S1 Appendix).

AI-mrEMVI⁺ patients had significantly shorter DFS than AI-mrEMVI^−^ patients in both the pathological complete response (pCR) and non-pCR subgroups (Fig 5E–F). Stratified analyses based on mrT stage, mrN stage, and tumor location consistently showed poorer DFS in the AI-mrEMVI^+^ group across all subgroups (Figs L and M in S1 Appendix). Furthermore, variations in slice thickness, spacing between slices, and pixel spacing showed minimal impact on the prognostic performance of the AI-mrEMVI model (Figs N and O in S1 Appendix).

## Discussion

In this study, we trained and validated an nnUNet-based segmentation model for identifying mrEMVI status in patients with rectal cancer. The model demonstrated strong classification performance and robust prognostic stratification across two independent external test cohorts, offering radiologists a consistent and objective tool to enhance mrEMVI detection. To the best of our knowledge, this study represents the first large-scale application of deep learning to voxel-level anatomical segmentation of mrEMVI using a radiologist-annotated multiplanar T2WI dataset. The model showed substantial agreement with senior radiologists. This agreement was numerically higher than that between senior and mid-level radiologists, although the difference was not statistically significant. By providing spatially interpretable segmentation outputs, the model has the potential to assist less experienced radiologists and standardize mrEMVI assessment across institutions.

mrEMVI is a crucial biomarker for predicting recurrence and metastasis in rectal cancer, playing a key role in clinical decision-making [6–13]. However, the lack of objective detection methods contributes to diagnostic variability, highlighting the need for more accurate and consistent assessment strategies. Several prior approaches have been proposed to address this limitation. Radiomics-based methods, such as the clinical-imaging model proposed by Shu et al. [37], have demonstrated promising results (AUC = 0.835); however, their dependence on manual segmentation, handcrafted feature extraction, and lack of external validation limit reproducibility and clinical scalability. Sasuga et al. [38] proposed a multi-label segmentation approach for rectal cancer T staging, demonstrating that deep learning can precisely delineate subtle tumor invasion beyond the rectal wall, a task that shares key characteristics with mrEMVI detection in terms of small-volume extramural extension and boundary ambiguity, suggesting that deep learning-based segmentation holds promise for structurally analogous tasks. Cai et al. [24] pioneered automated mrEMVI assessment using a deep learning pipeline, achieving an AUC of 0.76 in external validation. However, this approach relies on DWI as the primary segmentation channel, with T2WI downsampled to the DWI spatial domain as an auxiliary input, inevitably resulting in loss of high-resolution morphological detail. Furthermore, the pipeline provides only binary classification without precise spatial localization of mrEMVI regions. In contrast, our model operates on routinely acquired T2WI alone, achieving classification accuracies of 81.5% and 84.7% in two independent external test cohorts, comparing favorably with the classification-only pipeline, while additionally enabling spatial visualization and volumetric quantification of mrEMVI lesions. Comparative experiments showed that nnUNet v2 achieved the best segmentation performance in internal five-fold cross-validation and the highest overall classification accuracy in the combined external test cohorts. The underperformance of the comparator architectures is likely attributable to their fixed network configurations, which do not adapt to dataset-specific properties such as voxel spacing, patch size, and class imbalance, whereas nnUNet v2 automatically configures all preprocessing and training parameters based on the dataset fingerprint without manual tuning [30], reflecting its plug-and-play nature and enabling robust adaptation to the task-specific challenges of mrEMVI segmentation.

Although the model achieved a relatively low Dice coefficient for mrEMVI segmentation, its classification performance remained robust. This discrepancy is primarily attributable to the inherent characteristics of mrEMVI, which typically presents as a small-volume lesion with poorly defined boundaries. Minor deviations in boundary delineation can significantly affect the Dice coefficient due to its high sensitivity to spatial overlap. Inter-reader analysis also showed substantial boundary variability, particularly for dilated vascular structures, for which the inter-reader Dice score (0.321) was comparable to that of the model (0.335). Nevertheless, the model’s Dice score for intravascular tumor signals was lower than the inter-reader Dice (0.442 vs. 0.653), indicating room for further improvement. In contrast, classification tasks merely require the accurate determination of lesion presence or absence and are less reliant on precise spatial localization, thereby exhibiting greater robustness. In this study, the model accurately identified the presence of mrEMVI even in cases with low Dice coefficients (Fig D in S1 Appendix), further supporting the aforementioned observation. Similar observations have been reported in the imaging of small lesions such as breast microcalcifications and pulmonary nodules [39,40], where deep learning-based classification approaches have demonstrated robust performance despite the inherent challenges of small target detection. Furthermore, stratified analysis based on the median AI-mrEMVI volume demonstrated that the model achieved better segmentation performance in cases with larger lesions, suggesting that lesion size may influence segmentation accuracy. Therefore, despite the limited segmentation accuracy, the model demonstrated strong performance in prognostic stratification, underscoring its potential clinical relevance. Crucially, voxel-level segmentation provides clinical capabilities that extend beyond binary classification, including spatial visualization of involved vascular structures, volumetric quantification of mrEMVI burden, and the potential to inform treatment planning and clinical decision-making, as further supported by the prognostic value of AI-derived lesion volume demonstrated in this study. These outputs are uniquely enabled by segmentation and cannot be replicated by classification-only approaches.

In recent years, EMVI has been recognized as an important prognostic marker in rectal cancer. Accurate identification facilitates improved risk stratification, thereby helping to avoid overtreatment in low-risk patients and undertreatment in high-risk individuals. Hołdakowska et al. [41] reported that EMVI^+^ patients are unlikely to achieve a sustained clinical complete response (cCR) after nCRT, suggesting that these individuals may benefit more from surgery than from a watch-and-wait approach. Similarly, Bugg et al. [42] found a 3.7-fold increased risk of metachronous metastasis within one year in EMVI^+^ cases. Consistent findings were observed in our study. AI-mrEMVI^+^ patients had significantly shorter DFS and OS compared with AI-mrEMVI^−^ patients (3-year DFS rate: 62.3% vs. 84.9%; 5-year OS rate: 68.7% vs. 87.1%). Both univariable and multivariable analyses demonstrated that AI-mrEMVI status serves as a strong predictor of adverse clinical outcomes. Moreover, adding AI-mrEMVI to the baseline clinical model significantly improved prognostic discrimination and yielded numerically higher C-indices than incorporating expert-consensus mrEMVI, although the differences between the AI- and expert-based models were not statistically significant. Notably, beyond binary mrEMVI status, larger AI-identified lesion volumes were associated with worse survival. These findings highlight the potential value of segmentation for localizing lesions and generating quantitative imaging markers that may improve risk stratification. The prevalence of mrEMVI varied among the cohorts, with external test cohort 2 having a higher prevalence of approximately 41.6%, possibly reflecting the referral patterns of oncology-specialized hospitals. While this heterogeneity may affect external validity, it reflects real-world diversity and helps evaluate the model’s generalizability.

Approximately 15%–27% of rectal cancer patients have been reported to reach a pCR after nCRT [43–46]. For these patients, organ-preserving strategies such as “watch and wait” may be considered as alternatives to standard resection, provided that accurate patient selection is ensured [47–50]. In pCR patients with pre-treatment mrEMVI^+^, more intensive surveillance is warranted due to their higher risk of recurrence. Vascular invasion prior to nCRT suggests early systemic dissemination, and even with apparent regression, the risk of residual micrometastases and recurrence persists. This highlights the importance of considering baseline tumor biology in treatment planning. Our findings showed that the AI-mrEMVI status stratified recurrence risk in both the non-pCR and pCR populations, indicating that AI-mrEMVI^+^ remains a strong adverse prognostic marker, even among patients with pCR. Therefore, mrEMVI status should be incorporated into prognostic assessment, and patients with baseline mrEMVI^+^ should be monitored more closely.

## Limitations

Several limitations should be acknowledged. First, the model exhibited false positives due to misidentification of larger mesorectal vessels as mrEMVI. Second, this was a retrospective study involving only Chinese patients, which limits the generalizability of the findings to other populations. Third, variability in MRI scanners and imaging protocols may have affected model performance. Fourth, mrEMVI was annotated by a single radiologist, introducing potential bias. Fifth, all patients in the external test cohorts received nCRT, which precludes direct correlation between baseline MRI findings and postoperative pathology. Finally, while the AI model demonstrated reliable performance in mrEMVI detection and prognostic stratification, its boundary delineation accuracy remains limited and the visualized segmentation outputs should be interpreted as approximate spatial localization rather than precise boundary delineation. Although AI-derived mrEMVI lesion volume showed significant correlation with manually annotated volume, the wide limits of agreement indicate that AI-derived volumes are not perfectly interchangeable with manually annotated volumes for absolute measurement purposes; future prospective studies with standardized annotation protocols are warranted to further validate the clinical utility of volumetric mrEMVI assessment.

## Conclusion

In this study, we trained and validated a fully automated nnUNet-based model for mrEMVI segmentation, enabling automated identification of mrEMVI and potentially enhancing clinical diagnostic efficiency. This model could serve as a computer-aided diagnostic tool in the future and could be integrated into PACS to assist with second reading, case triage, and quality control alerts, thereby improving the accuracy and consistency of mrEMVI identification. Additionally, the study highlights that incorporating AI-based mrEMVI assessment into prognostic evaluation can provide valuable insights for stratifying patients’ recurrence risks and optimizing personalized treatment strategies.

## Supporting information

S1 Appendix Supplementary methods, figures and tables. Text A–D, Fig A–O, Table A–F. Text A. Detailed information on the training and external test cohorts. Text B. Data standards. Text C. Detailed training procedure. Text D. Detailed inference procedure. Fig A. Patient enrollment flowchart for the training and external testing of the AI-mrEMVI model. Fig B. Flowchart of MRI marker evaluation. Fig C. Construction of the segmentation training dataset and five-fold cross-validation workflow for the AI-mrEMVI model. Fig D. The performance of the AI-mrEMVI model across different Dice scores. Fig E. Representative comparisons of AI-mrEMVI model predictions and radiologist annotations in typical, ambiguous, and misclassified cases. Fig F. Site-stratified confusion matrices and performance metrics of the AI-mrEMVI model in external test cohort 2. Fig G. The prognostic value of AI-mrEMVI status. Fig H. Bootstrap-derived distributions of C-index for DFS and OS across three models. Fig I. Kaplan-Meier survival curves for discordant cases between AI-mrEMVI and radiologist ground truth assessment. Fig J. The prognostic value of AI-mrEMVI lesion volume in external test cohort 2. Fig K. Correlation and agreement between AI-derived and manually annotated mrEMVI lesion volumes in true-positive cases. Fig L. Kaplan-Meier curves for DFS according to AI-mrEMVI status in different mrT and mrN staging subgroups. Fig M. Kaplan-Meier curves for DFS according to AI-mrEMVI status in different tumor location subgroups. Fig N. Kaplan-Meier curves for DFS according to AI-mrEMVI status in different subgroups of MR T2WI images with varying slice thicknesses and spacing between slices. Fig O. Kaplan-Meier curves for DFS according to AI-mrEMVI status in different subgroups of MR T2WI images with varying pixel spacing. Table A. MRI acquisition parameters across the participating centers. Table B. Segmentation performance comparison across deep learning architectures in internal five-fold cross-validation. Table C. Classification performance comparison across deep learning architectures on the combined external test cohorts. Table D. Delta C-index and 95% confidence intervals for model comparisons in disease-free survival (DFS) and overall survival (OS). Table E. Univariable and multivariable analyses of OS. Table F. Schoenfeld residual test results for proportional hazards assumption in multivariable Cox regression models of DFS and OS in two external test cohorts.
(PDF)
