# Supplementary material for: Artificial intelligence for evaluation of magnetic resonance imaging-detected extramural vascular invasion in rectal cancer
Source: PLOS Digit Health. 2026 Oct 1;5(10):e0001763. doi: 10.1371/journal.pdig.0001763 (PMC13630187; doi:10.1371/journal.pdig.0001763)
Supplement: Supplementary file 1 [file pdig.0001763.s001.pdf]

S1 Appendix

Text A. Detailed information on the training and external test cohorts

During the training phase of the model, a total of 1830 patients were included in the study, originating from four hospitals: Shanxi Cancer Hospital (SXCH, Taiyuan, China), Sun Yat-sen University Cancer Center (SYSUCC, Guangzhou, China), Yunnan Cancer Hospital (YNCH, Kunming, China), and the First Affiliated Hospital of Wenzhou Medical University (WZH, Wenzhou, China) from February 2009 to March 2020, forming the training cohort. The treatment methods for these patients were not strictly defined; however, it is certain that all underwent standard total mesorectal excision surgery and had pelvic MRI scans performed prior to treatment. In order to assess the predictive performance of the model, this study included 671 patients who underwent radical surgery after neoadjuvant chemoradiotherapy as the two external test cohorts. The data of these patients were gathered between June 2012 and November 2018 at Guangdong Provincial People's Hospital (GDPH, Guangzhou, China), Sun Yat-sen University Sixth Affiliated Hospital (SYSU6, Guangzhou, China), Shanxi Cancer Hospital (SXCH, Taiyuan, China), and Fudan University Cancer Center (FDCC, Shanghai, China). Notably, the SXCH sub-cohort within the training cohort comprised exclusively patients who underwent direct surgery without nCRT. Patients from the remaining three centers (SYSUCC, YNCH, and WZH) included both patients who underwent direct surgery and those who received nCRT prior to surgery. This mixed inclusion strategy was adopted to maximize the number of eligible patients and enhance model generalizability across heterogeneous clinical presentations and treatment backgrounds.

Text B. Data standards

The inclusion criteria for the training cohort were as follows: (1) age ⩾ 18 years and histopathologically confirmed rectal adenocarcinoma; (2) treatment with standard total mesorectal

excision; (3) MRI satisfying the prespecified spatial-resolution requirements, with an in-plane pixel spacing ≤0.7 mm and a slice thickness ≤5 mm; and (4) complete clinical and pathological data, with at least one diagnostic-quality axial, coronal, or sagittal T2-weighted imaging (T2WI) series available for analysis. The exclusion criteria were as follows: (1) absence of all axial, coronal, and sagittal T2WI series; (2) insufficient diagnostic quality of all available T2WI series; or (3) incomplete clinical or pathological data.

The inclusion criteria for the external test cohorts were as follows: (1) age ≥18 years and an initial diagnosis of locally advanced rectal cancer (cT3–4 and/or cN1–2, or mrEMVI-positive; cM0) before treatment; (2) standard total mesorectal excision performed within 8–12 weeks after completion of standard neoadjuvant chemoradiotherapy (nCRT); and (3) high-resolution rectal MRI performed within 2 weeks before initiation of nCRT, with at least one diagnostic-quality axial, coronal, or sagittal T2WI series available. The exclusion criteria were as follows: (1) concurrent malignancy, metastatic disease, or prior cancer treatment at the initial consultation; (2) nonstandard or incomplete nCRT, or neoadjuvant chemotherapy or radiotherapy alone; (3) palliative treatment for any reason; (4) unavailable pathological response assessment of the surgical specimen; (5) absence of all axial, coronal, and sagittal T2WI series or insufficient diagnostic quality of all available T2WI series; or (6) missing or incomplete clinical follow-up data.

For both the training and external test cohorts, acquisition of T2WI in all three planes was not required. Patients with only one or two available planes remained eligible, provided that at least one plane was of diagnostic quality. Each available plane was processed independently, and missing planes were neither imputed nor reconstructed.

Text C. Detailed training procedure

3.1 Computing Environment and Software

All model training and inference were conducted on a workstation equipped with an Intel(R) Core™ i9-10980XE CPU @ 3.00GHz and an NVIDIA GeForce RTX 4090 GPU (24GB VRAM), running Ubuntu 24.04.1 LTS. All deep learning workflows were implemented using PyTorch within the nnUNet v2 framework.

3.2 Training Data Preparation and Preprocessing

The overall training cohort comprised 1,830 patients. For segmentation model training, all 499 $mrEMVI^+$ patients and 247 randomly selected $mrEMVI^-$ patients were included, yielding a segmentation training dataset of 746 patients with an approximate positive-to-negative ratio of 2:1. These patients contributed 1,289 manually annotated plane-specific T2WI volumes, which served as the ground-truth dataset.

Prior to training, all MR images underwent standardized preprocessing automatically determined by the nnUNet v2 framework based on the dataset fingerprint, including: (1) non-zero cropping to remove irrelevant background; (2) resampling to an isotropic in-plane resolution of 0.3516 mm with a slice thickness of 3.3 mm to address voxel spacing inconsistencies across patients; and (3) z-score normalization using the mean (265.14) and standard deviation (296.61) computed from all foreground voxels in the training dataset. Intensity values were additionally clipped to the 0.5th–99.5th percentile range of foreground voxels (42.0–2032.0) to suppress outlier intensity values.

All available T2WI planes (axial, coronal, and/or sagittal) were treated as separate input cases within the standard nnUNet framework and processed independently. Missing planes were neither imputed nor reconstructed, and no custom multi-plane fusion module was implemented.

During training, a patch-based sampling strategy was adopted. Patch size was determined based on

the dataset median image size, capped at 128×128×128 if exceeded.

3.3 Data Augmentation

To improve model robustness and generalizability, online data augmentation was applied during training with a probability of 0.5. The augmentation pipeline included: elastic deformation (alpha: 0–900, sigma: 9–13), random rotation (−30° to 30°), scaling (0.7–1.4×), gamma correction (γ: 0.7–1.5), mirroring, Gaussian noise (standard deviation: 0–0.1), and Gaussian blur. These transformations increased the diversity of training samples and reduced the risk of overfitting.

3.4 Network Architecture and Training Configuration

The segmentation model was trained using the standard nnUNet v2 framework with the 3D full-resolution U-Net architecture. The network replaced standard ReLU activations with LeakyReLU ($F(x) = \max(ax,\ x)\ , a = 0.01$) and used Instance Normalization instead of Batch Normalization to improve performance at small batch sizes. The initial number of feature channels was 32, doubling at each encoder stage up to a maximum of 320.

The model input dimension was $X \epsilon R^{(B,N,D,H,W)}$ and output dimension was $Y \epsilon R^{(B,M,D,H,W)}$, where N denotes the number of modalities (set to 1) and M denotes the number of segmentation classes (set to 4: background, Label 0; primary tumor, Label 1; intravascular tumor signals, Label 2; and dilated vascular structures, Label 3).

The loss function combined Dice loss and cross-entropy loss, defined as $L_{total} = L_{dice} + L_{ce}$. Model optimization used Nesterov momentum stochastic gradient descent with a momentum coefficient of 0.99.

Training was conducted for a maximum of 1,000 epochs, with each epoch consisting of 250 mini-

batches. The initial learning rate was set to 0.01. The batch size was automatically determined by the nnUNet v2 framework based on available GPU memory and input image dimensions, resolving to 2 in this study given the RTX 4090 GPU with 24GB VRAM.

The learning rate was decayed by a factor of 5 if the training loss did not decrease by at least 5×10^{-3} within the past 30 epochs. Training was automatically terminated when the validation loss did not decrease by more than 5×10^{-3} over the last 60 epochs and the current learning rate was below 10^{-6}.

3.5 Cross-validation and Model Ensemble

Five-fold cross-validation was performed at the plane-specific volume level to evaluate model performance and improve robustness. The segmentation training dataset of 1,289 plane-specific T2WI volumes was partitioned into five folds. In each iteration, one-fold served as the validation set, while the remaining four folds were used for training, ensuring that each plane-specific volume was used for validation exactly once. Across folds 0–4, the training subsets comprised 662/369, 672/359, 671/360, 665/366, and 670/362 mrEMVI^{+}/mrEMVI^{-} volumes, respectively, while the corresponding validation subsets contained 258, 258, 258, 258, and 257 volumes. The five trained models were combined into an ensemble for subsequent inference, with their predictions aggregated by voxel-wise averaging of the softmax probability maps.

Text D. Detailed inference procedure

4.1 System Requirements

The released model package can be executed on a workstation or server running Linux, provided that PyTorch and nnUNet are properly installed. In this study, inference was performed on a workstation equipped with an NVIDIA GeForce RTX 4090 GPU with 24 GB VRAM. CPU-only inference is feasible

but substantially slower.

4.2 Software Installation and Environment Setup

Model inference was conducted using the nnUNet framework implemented in PyTorch. Users should install a compatible Python environment and the required dependencies according to the official nnUNet documentation. After installation, the model package should be placed under the appropriate nnUNet results directory so that the trained fold checkpoints can be recognized automatically by the framework. Environment configuration should be verified before running inference.

4.3 Input Data Preparation

For inference, each available imaging plane was provided as an independent NIfTI file in `.nii.gz` format, following the standard nnUNet naming convention: `<case_identifier>_0000.nii.gz`. Each available axial, coronal, and/or sagittal T2WI plane was treated as a separate input case and processed independently. Missing planes were neither imputed nor reconstructed. The model generated a corresponding segmentation mask for each available plane, and patient-level AI-mrEMVI status was determined by aggregating predictions across all available planes. A patient was classified as AI-mrEMVI+ if at least one voxel labeled as mrEMVI (Label 2 or Label 3) was detected in any available output mask.

The model was trained using multiplanar T2-weighted MR images. Therefore, input images should be consistent with the imaging modality used during training to ensure valid model predictions.

4.4 Preprocessing During Inference

The preprocessing pipeline during inference was identical to that used for training and was automatically executed by nnUNet. This pipeline included non-zero cropping, resampling to the target

voxel spacing defined during model planning, and z-score normalization. Applying the same preprocessing steps during inference ensured consistency between the training and deployment stages.

4.5 Inference Execution

Inference can be performed using either a single-fold model or a five-fold ensemble model. The ensemble approach is recommended because it generally provides more stable and robust predictions.

A representative nnUNet command for five-fold ensemble inference is shown below:

```
nnUNetv2_predict \
  -i /path/to/input_folder \
  -o /path/to/output_folder \
  -d DATASET_ID \
  -c 3d_fullres \
  -f 0 1 2 3 4
```

For single-fold inference, the fold list can be restricted to one fold only, for example:

```
nnUNetv2_predict \
  -i /path/to/input_folder \
  -o /path/to/output_folder \
  -d DATASET_ID \
  -c 3d_fullres \
  -f 0
```

In both settings, preprocessing, patch-based prediction, and output generation are handled automatically by nnUNet.

4.6 Ensemble Strategy

For five-fold inference, predictions from the five trained models were combined using the standard nnUNet ensemble strategy. Specifically, the softmax probability maps generated by each fold were averaged voxel-wise, and the final segmentation label for each voxel was determined by selecting the class with the highest mean probability. This approach reduces prediction variance and improves segmentation robustness compared with any single-fold model.

4.7 Output Files and Interpretation

The output of the model is a segmentation map in NIfTI format. Each voxel in the output image is assigned an integer label corresponding to a predefined class. In the present model, the labels were defined as follows: background (Label 0); primary tumor (Label 1); mrEMVI regions comprising intravascular tumor signals (Label 2) and dilated vascular structures (Label 3). For patient-level classification, a patient was considered AI-mrEMVI$^+$ if at least one voxel assigned Label 2 or Label 3 was detected in any available plane-specific output segmentation map. A patient was classified as AI-mrEMVI$^-$ if no such voxel was detected in any available output map.

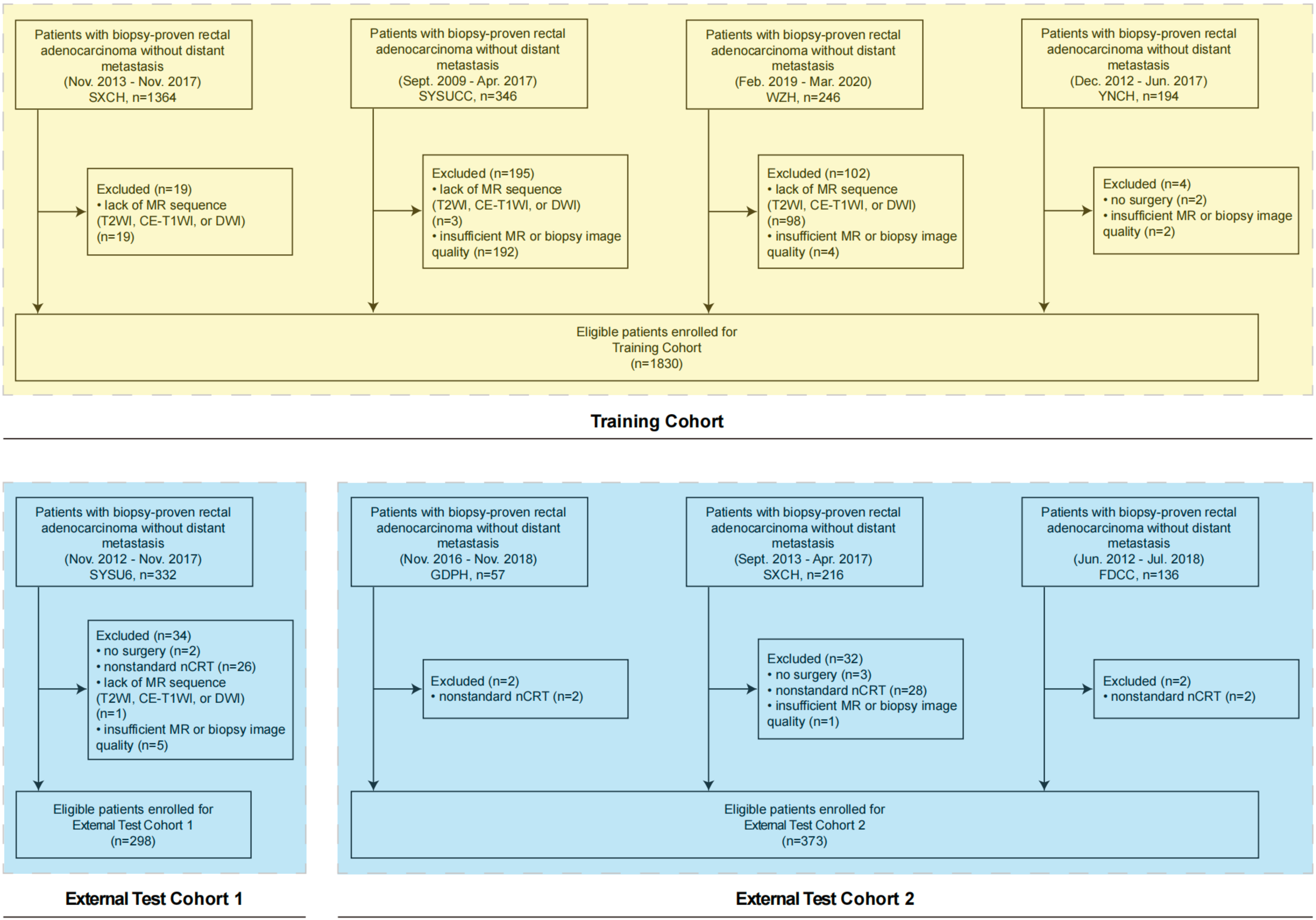

Patients with biopsy-proven rectal adenocarcinoma without distant metastasis (Nov. 2013 - Nov. 2017) SXCH, n=1364
Excluded (n=19) • lack of MR sequence (T2WI, CE-T1WI, or DWI) (n=19)
Patients with biopsy-proven rectal adenocarcinoma without distant metastasis (Sept. 2009 - Apr. 2017) SYSUCC, n=346
Excluded (n=195) • lack of MR sequence (T2WI, CE-T1WI, or DWI) (n=3) • insufficient MR or biopsy image quality (n=192)
Patients with biopsy-proven rectal adenocarcinoma without distant metastasis (Feb. 2019 - Mar. 2020) WZH, n=246
Excluded (n=102) • lack of MR sequence (T2WI, CE-T1WI, or DWI) (n=98) • insufficient MR or biopsy image quality (n=4)
Patients with biopsy-proven rectal adenocarcinoma without distant metastasis (Dec. 2012 - Jun. 2017) YNCH, n=194
Excluded (n=4) • no surgery (n=2) • insufficient MR or biopsy image quality (n=2)
Eligible patients enrolled for Training Cohort (n=1830)
Training Cohort
Patients with biopsy-proven rectal adenocarcinoma without distant metastasis (Nov. 2012 - Nov. 2017) SYSU6, n=332
Excluded (n=34) • no surgery (n=2) • nonstandard nCRT (n=26) • lack of MR sequence (T2WI, CE-T1WI, or DWI) (n=1) • insufficient MR or biopsy image quality (n=5)
Eligible patients enrolled for External Test Cohort 1 (n=298)
Patients with biopsy-proven rectal adenocarcinoma without distant metastasis (Nov. 2016 - Nov. 2018) GDPH, n=57
Excluded (n=2) • nonstandard nCRT (n=2)
Patients with biopsy-proven rectal adenocarcinoma without distant metastasis (Sept. 2013 - Apr. 2017) SXCH, n=216
Excluded (n=32) • no surgery (n=3) • nonstandard nCRT (n=28) • insufficient MR or biopsy image quality (n=1)
Patients with biopsy-proven rectal adenocarcinoma without distant metastasis (Jun. 2012 - Jul. 2018) FDCC, n=136
Excluded (n=2) • nonstandard nCRT (n=2)
Eligible patients enrolled for External Test Cohort 2 (n=373)
External Test Cohort 1
External Test Cohort 2

Fig A. Patient enrollment flowchart for the training and external testing of the AI-mrEMVI model. In the training cohort patients were consecutively enrolled from four independent institutions: SXCH (Shanxi Cancer Hospital); SYSUCC (Sun Yat-sen University Cancer Center); YNCH (Yunnan Cancer Hospital); and WZH (The First Affiliated Hospital of Wenzhou Medical University). In the external test cohort 1, patients were recruited from one hospital: SYSU6 (The Sixth Affiliated Hospital of Sun Yat-sen University). In the external test cohort 2, patients were recruited from three hospitals: GDPH (Guangdong Provincial People's Hospital); SXCH (Shanxi Cancer Hospital); and FDCC (Fudan University Cancer Center). AI, artificial intelligence. mr, magnetic resonance. EMVI, extramural vascular invasion.

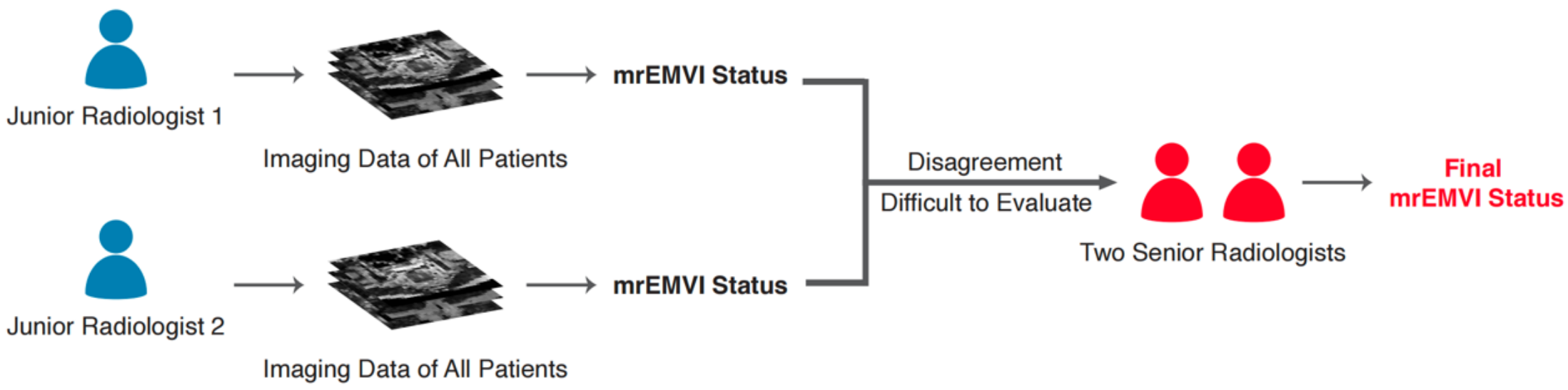

Fig B. Flowchart of MRI marker evaluation. Baseline mrEMVI status was independently assessed by two junior radiologists (each with 5 years of experience) for all patients in the training and external test cohorts. Cases with discrepant assessments or those deemed difficult to evaluate were referred to two senior radiologists (each with over 20 years of experience), who jointly reviewed these cases and reached a final consensus through discussion. mr, magnetic resonance; EMVI, extramural vascular invasion.

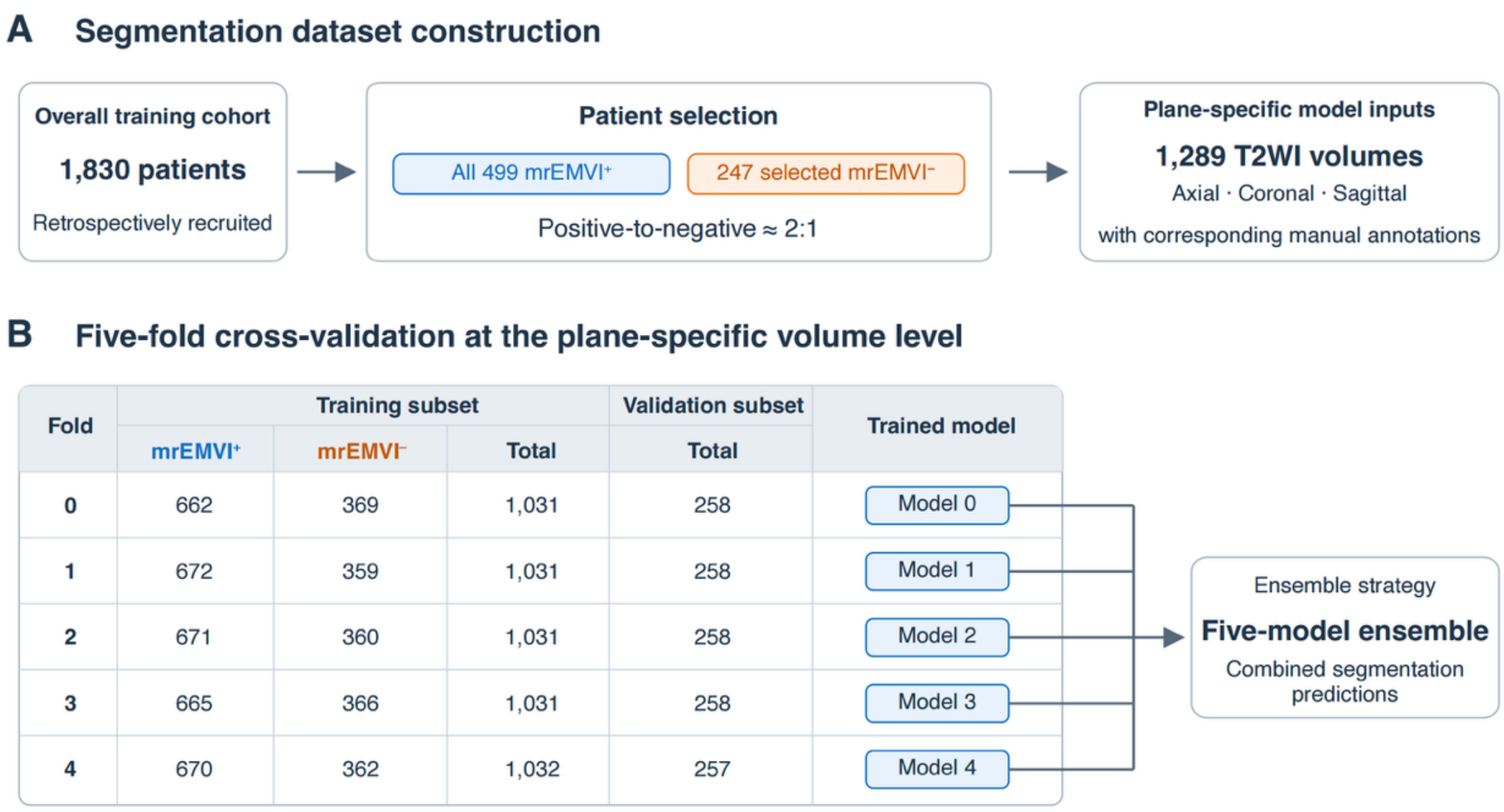

Fig C. Construction of the segmentation training dataset and five-fold cross-validation workflow for the AI-mrEMVI model. (A) All 499 mrEMVI$^+$ patients and 247 selected mrEMVI$^-$ patients from the overall training cohort of 1,830 patients were included, yielding a segmentation training dataset of 746 patients. These patients contributed 1,289 manually annotated plane-specific T2WI volumes in the axial, coronal, and sagittal planes. (B) Five-fold cross-validation was performed at the plane-specific volume level. The exact numbers of mrEMVI$^+$ and mrEMVI$^-$ volumes in each training subset are shown. Each fold generated a separately trained model, and predictions from the five models were combined using an ensemble strategy. mr, magnetic resonance; EMVI, extramural venous invasion; T2WI, T2-weighted imaging.

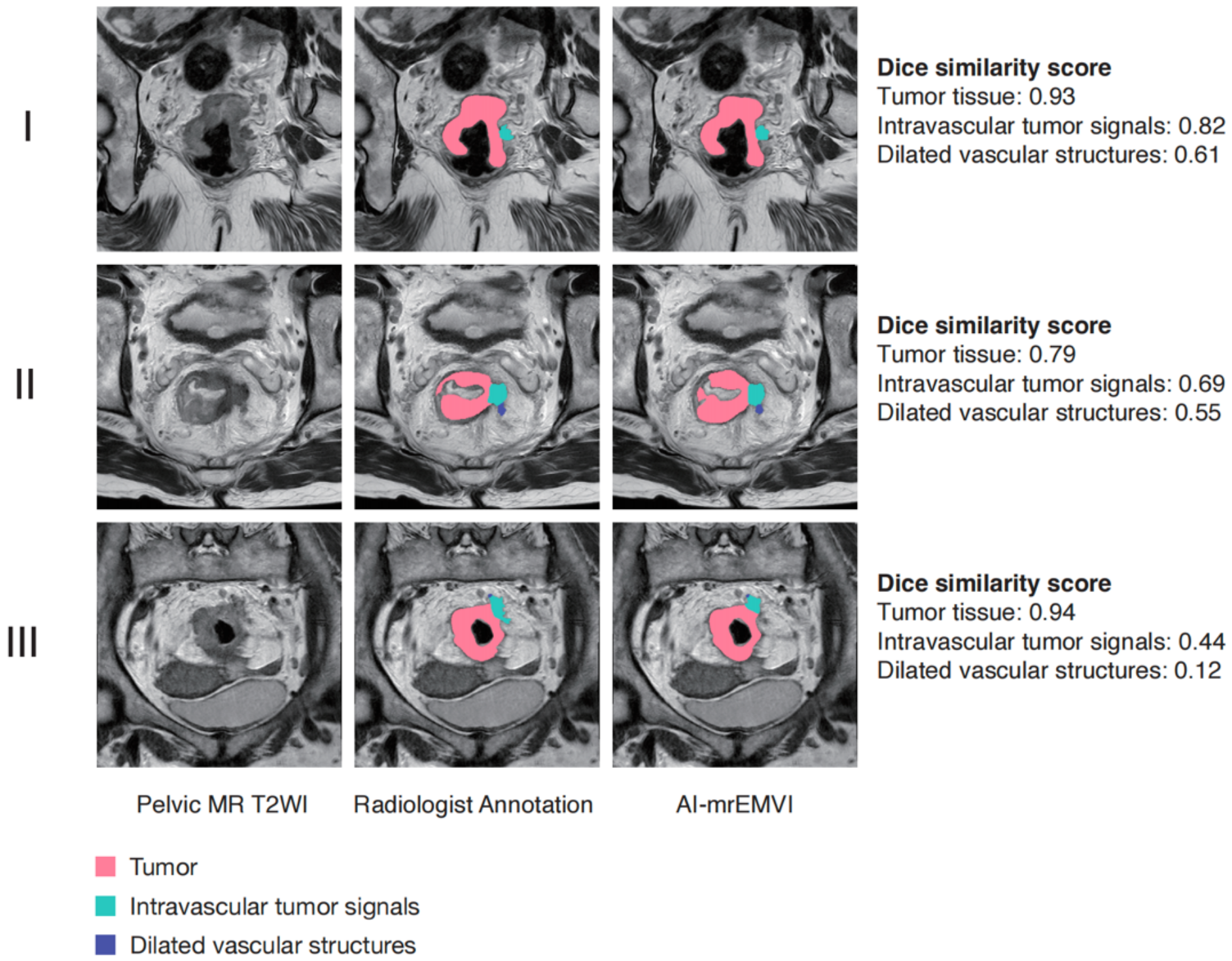

Fig D. The performance of the AI-mrEMVI model across different Dice scores. The rows **I–III** present various segmentation cases from all external test cohorts. From left to right, they show T2WI, manual delineation by physicians, and the results identified by the AI-mrEMVI model. Even though some cases have low Dice scores, the model is still able to correctly identify AI-mrEMVI^{+} cases. AI, artificial intelligence; mr, magnetic resonance; EMVI, extramural vascular invasion; T2WI, T2-weighted images.

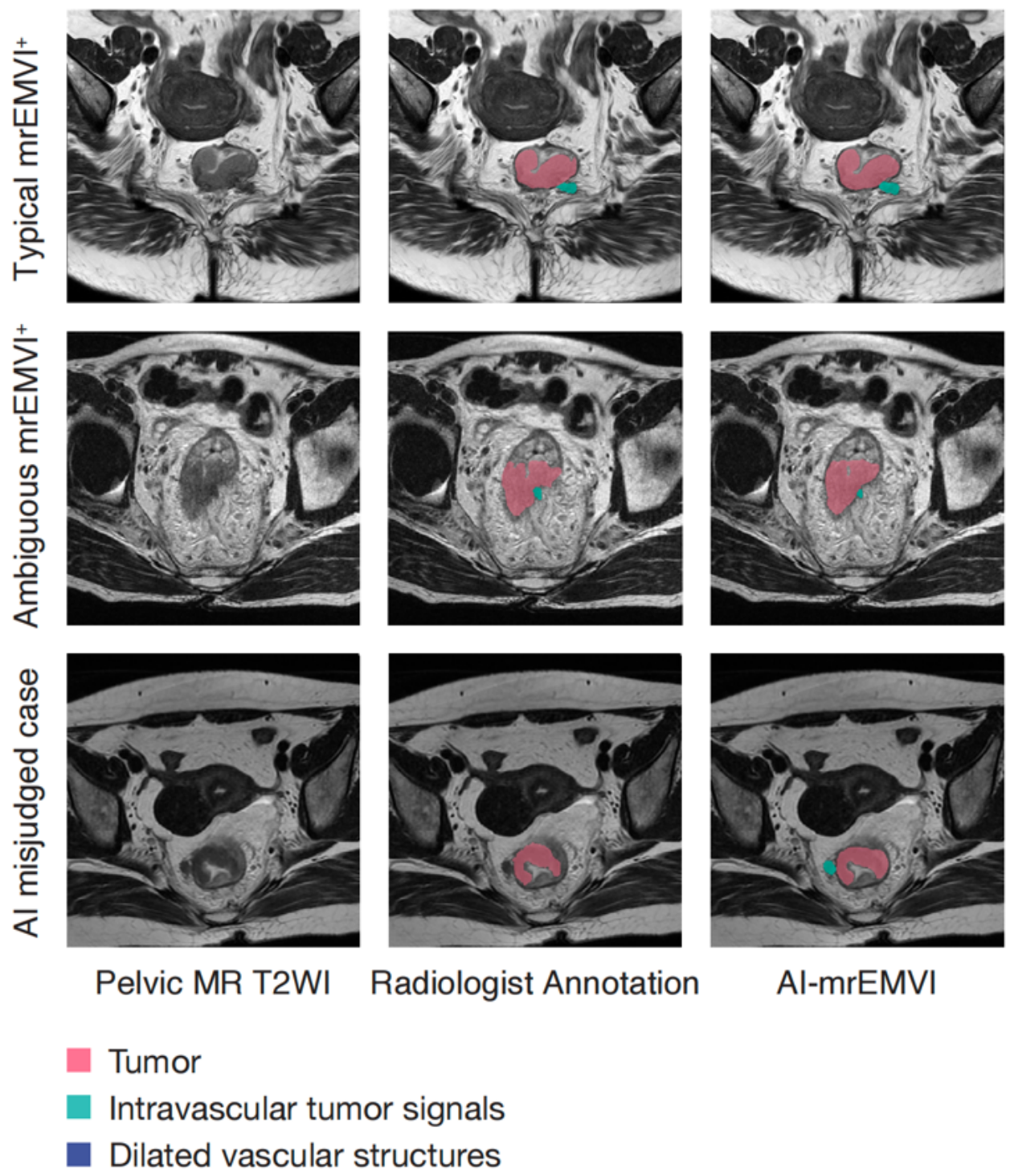

Fig E. Representative comparisons of AI-mrEMVI model predictions and radiologist annotations in typical, ambiguous, and misclassified cases. In the ambiguous mrEMVI$^+$ case, the tumor followed an S-shaped trajectory from the anterosuperior to the posteroinferior direction, with the mrEMVI lesion located precisely at the turning point. Initial assessments by junior and intermediate radiologists yielded substantial disagreement regarding the mrEMVI status. Following a joint review by two senior radiologists, the case was ultimately determined to be mrEMVI$^+$. Notably, the AI model successfully identified and segmented the lesion in this anatomically challenging location, demonstrating strong robustness. In the misclassified case, the AI model incorrectly identified a metastatic lymph node closely adjacent to the primary tumor as an mrEMVI$^+$ region. From left to right, each panel shows the T2WI, manual annotations by radiologists, and segmentation results generated by the AI-mrEMVI model. Color overlays: red indicates the primary tumor tissue; green indicates intravascular tumor signals; purple indicates dilated vascular structures. AI, artificial intelligence; mr, magnetic resonance; EMVI, extramural vascular invasion; T2WI, T2-weighted imaging.

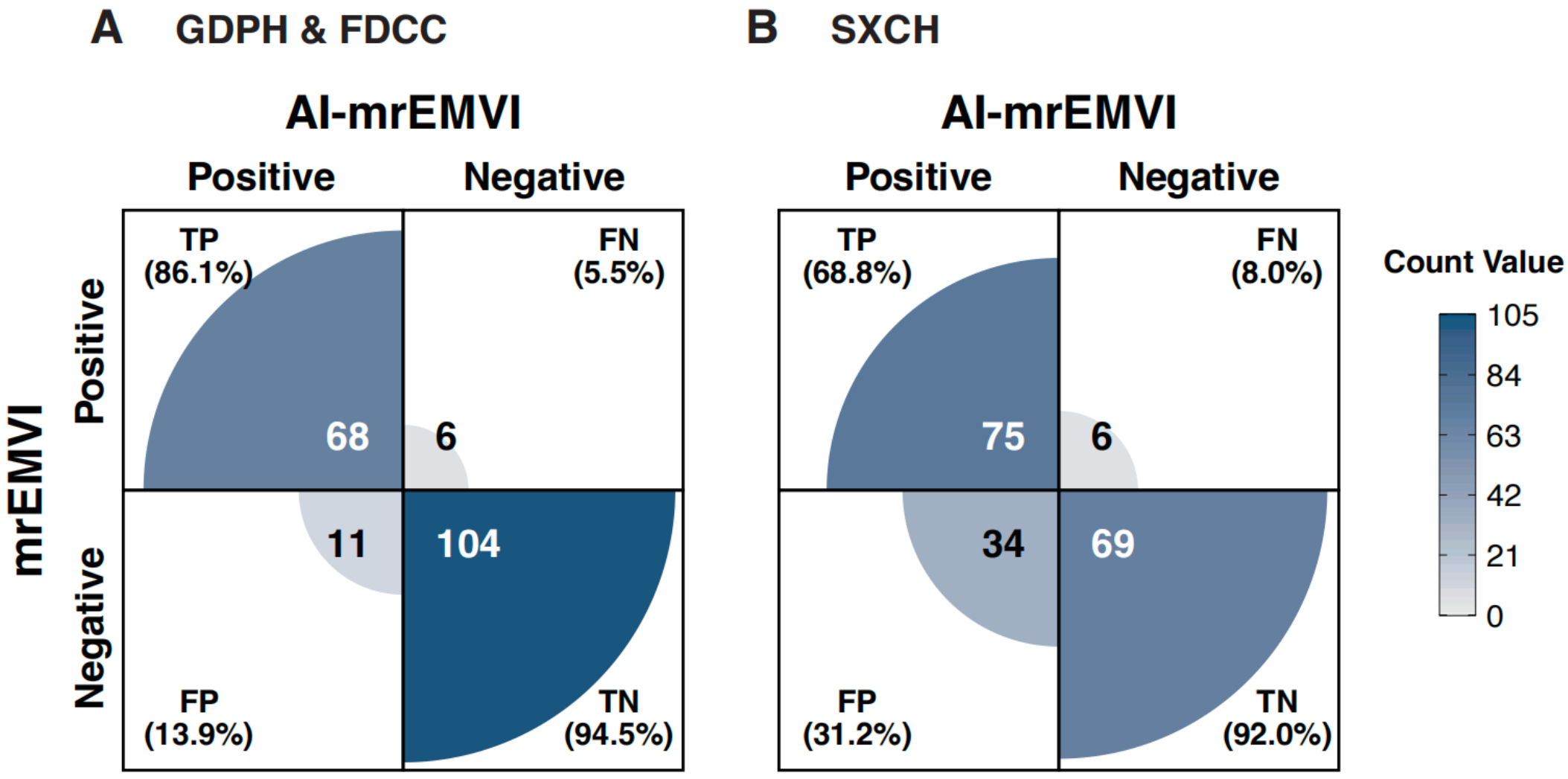

C Classification Performance Metrics of the Model

	Accuracy (95% CI)	Sensitivity (95% CI)	Specificity (95% CI)
**External Test Cohort 2**	84.7% (80.7%–88.0%)	92.3% (87.0%–95.5%)	79.4% (73.5%–84.2%)
**GDPH & FDCC**	91.0% (86.1%–94.3%)	91.9% (83.4%–96.2%)	90.4% (83.7%–94.6%)
**SXCH**	78.3% (71.8%–83.6%)	92.6% (84.8%–96.6%)	67.0% (57.4%–75.3%)

	PPV (95% CI)	NPV (95% CI)	AUC (95% CI)
**External Test Cohort 2**	76.1% (69.5%–81.6%)	93.5% (89.0%–96.3%)	0.858 (0.824–0.892)
**GDPH & FDCC**	86.1% (76.8%–92.0%)	94.5% (88.6%–97.5%)	0.912 (0.870–0.953)
**SXCH**	68.8% (59.6%–76.7%)	92.0% (83.6%–96.3%)	0.798 (0.744–0.852)

Fig F. Site-stratified confusion matrices and performance metrics of the AI-mrEMVI model in External Test Cohort 2. (A) Confusion matrix for the GDPH & FDCC sub-cohort. **(B)** Confusion matrix for the SXCH sub-cohort. **(C)** Classification performance metrics stratified by institution. AI, artificial intelligence; mr, magnetic resonance; EMVI, extramural vascular invasion; PPV, Positive Predictive Value; NPV, Negative Predictive Value; AUC, Area Under the ROC Curve.

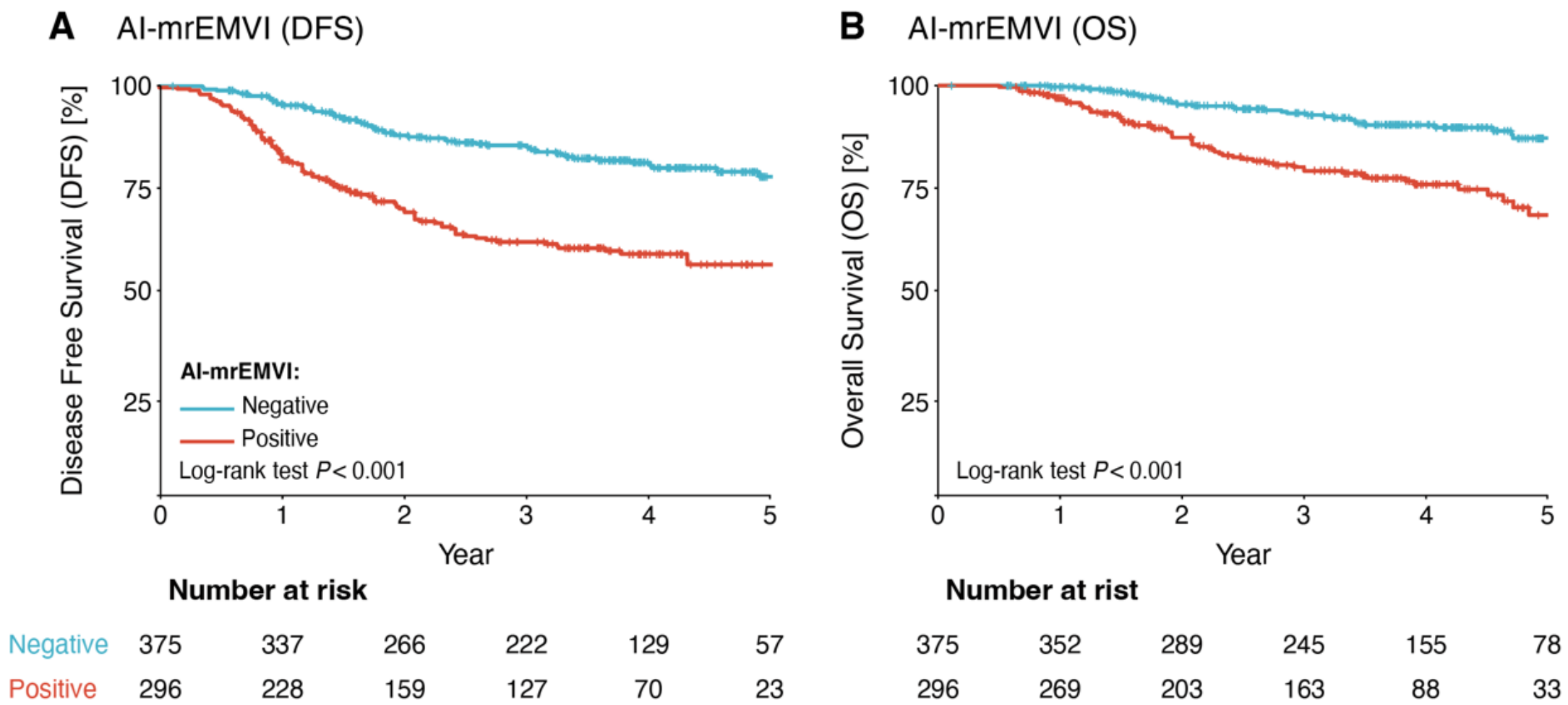

Fig G. The prognostic value of AI-mrEMVI status. (A) The Kaplan-Meier survival curves for DFS of patients in two external test cohorts. **(B)** Kaplan–Meier survival curves for OS of patients in two external test cohorts. AI, artificial intelligence; mr, magnetic resonance; EMVI, extramural vascular invasion; DFS, disease-free survival; OS, overall survival.

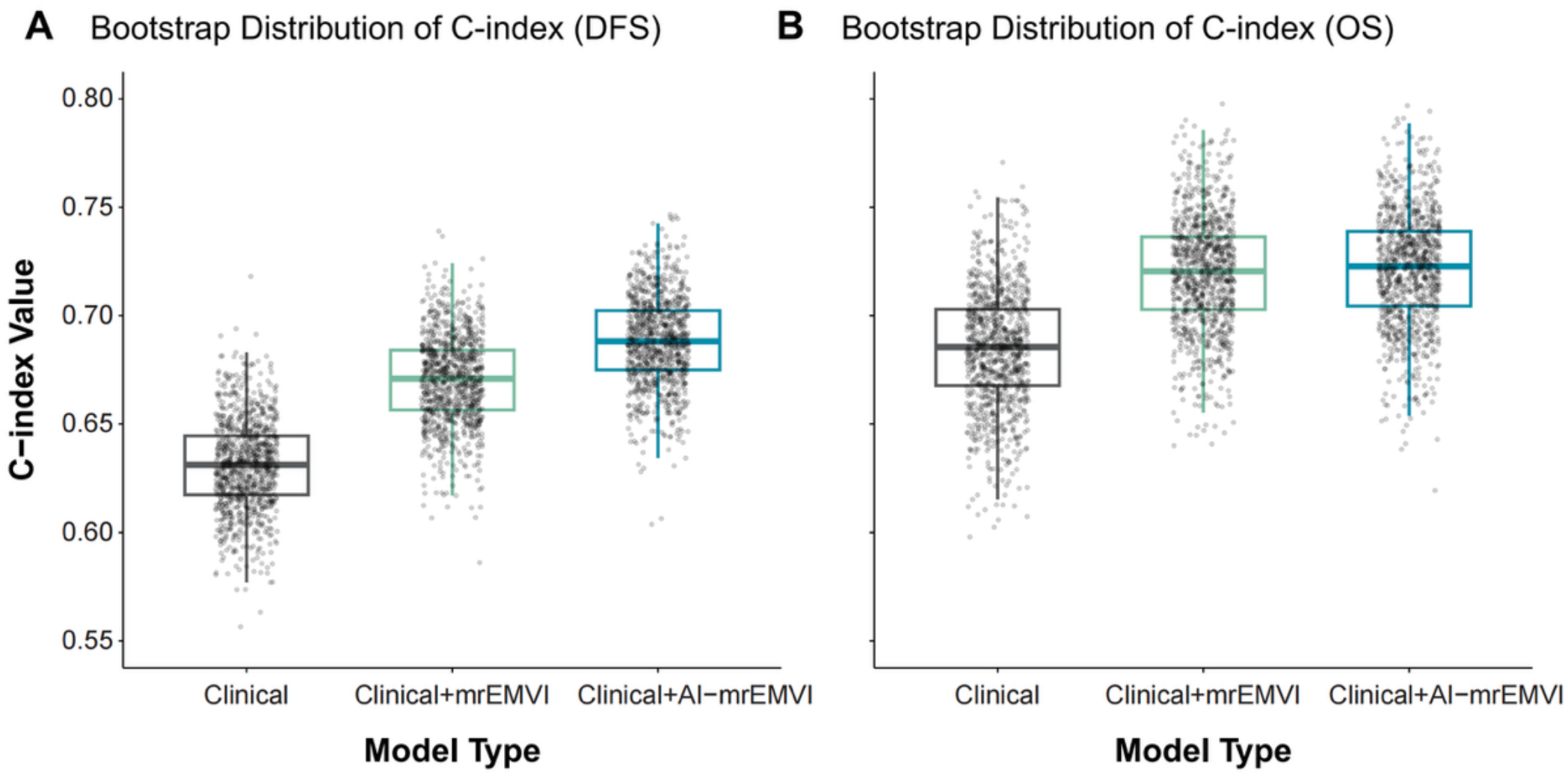

Fig H. Bootstrap-derived distributions of C-index for DFS and OS across three models. (A) Distribution of C-index values for DFS, and **(B)** for OS, obtained from 1000 bootstrap resamples. Boxplots compare three Cox proportional hazards models: the clinical model (including mrT stage, mrN stage, CEA level, and tumor location), the clinical + radiologist-assessed mrEMVI model, and the clinical + AI-mrEMVI model. DFS, disease-free survival; OS, overall survival; AI, artificial intelligence; mr, magnetic resonance; EMVI, extramural vascular invasion; CEA, carcinoembryonic antigen.

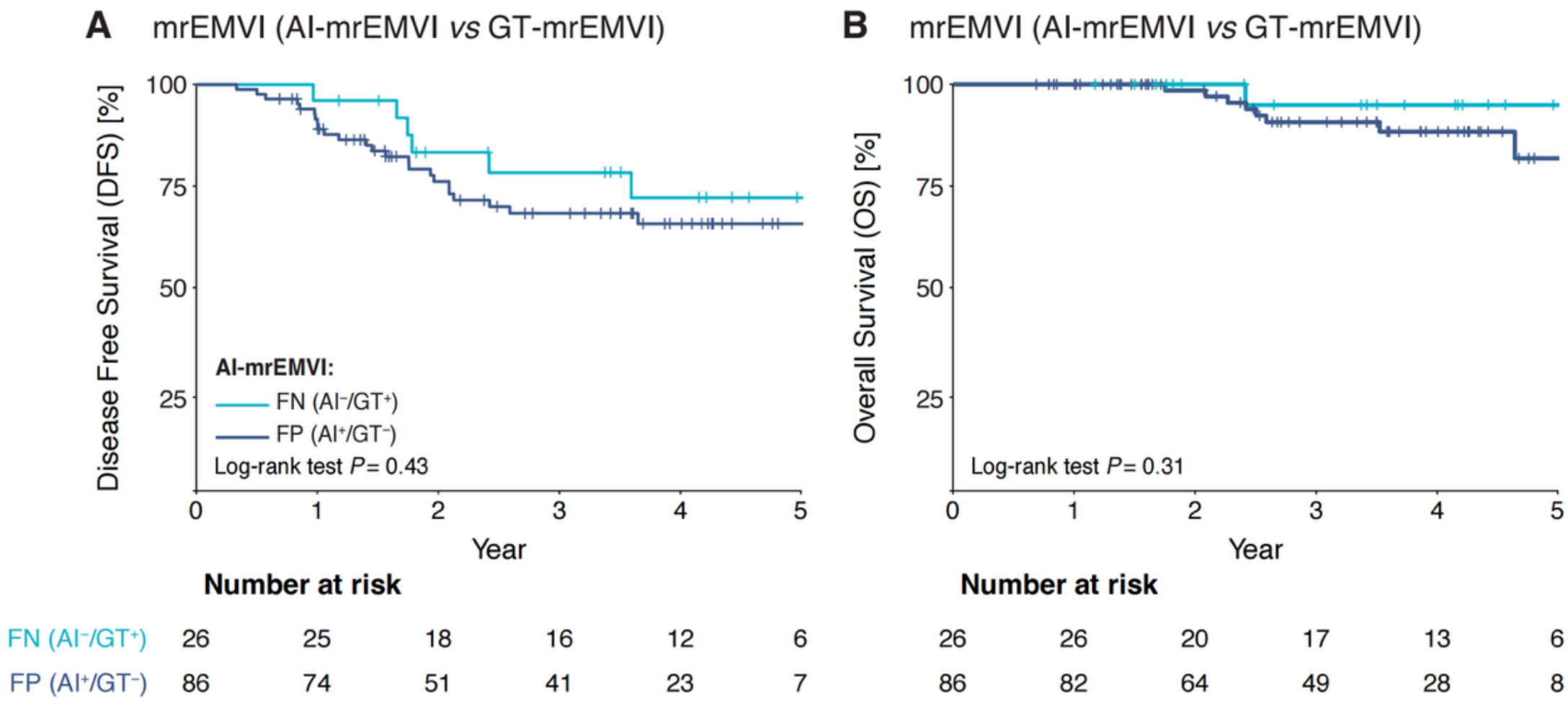

Fig I. Kaplan-Meier survival curves for discordant cases between AI-mrEMVI and radiologist ground truth assessment. (A) Disease-free survival (DFS) and **(B)** overall survival (OS) comparing patients with false-negative AI assessments (FN: AI-mrEMVI^{-}/GT-mrEMVI^{+}, n = 26) and false-positive AI assessments (FP: AI-mrEMVI^{+}/GT-mrEMVI^{-}, n = 86). AI, artificial intelligence; mr, magnetic resonance; EMVI, extramural vascular invasion; GT, radiologist ground truth; DFS, disease-free survival; OS, overall survival; FN, false negative; FP, false positive.

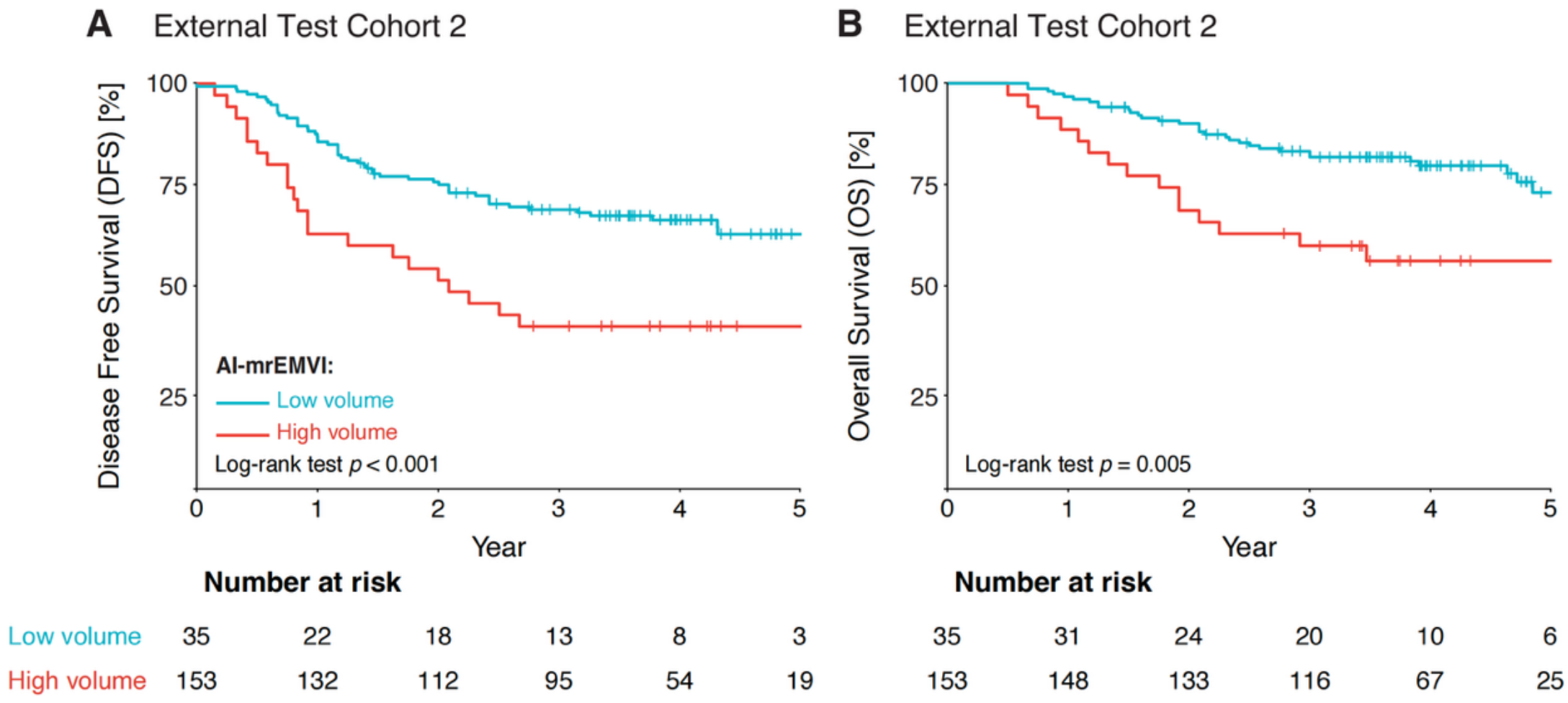

Fig J. The prognostic value of AI-mrEMVI lesion volume in External Test Cohort 2. (A) Kaplan–Meier survival curves for DFS stratified by lesion volume using an optimal cutoff of 2329.16 mm^3 derived from External Test Cohort 1 ($p < 0.001$). **(B)** Kaplan–Meier survival curves for OS stratified by lesion volume using an optimal cutoff of 2729.58 mm^3 derived from External Test Cohort 1 ($p = 0.005$). AI, artificial intelligence; mr, magnetic resonance; EMVI, extramural vascular invasion; DFS, disease-free survival; OS, overall survival.

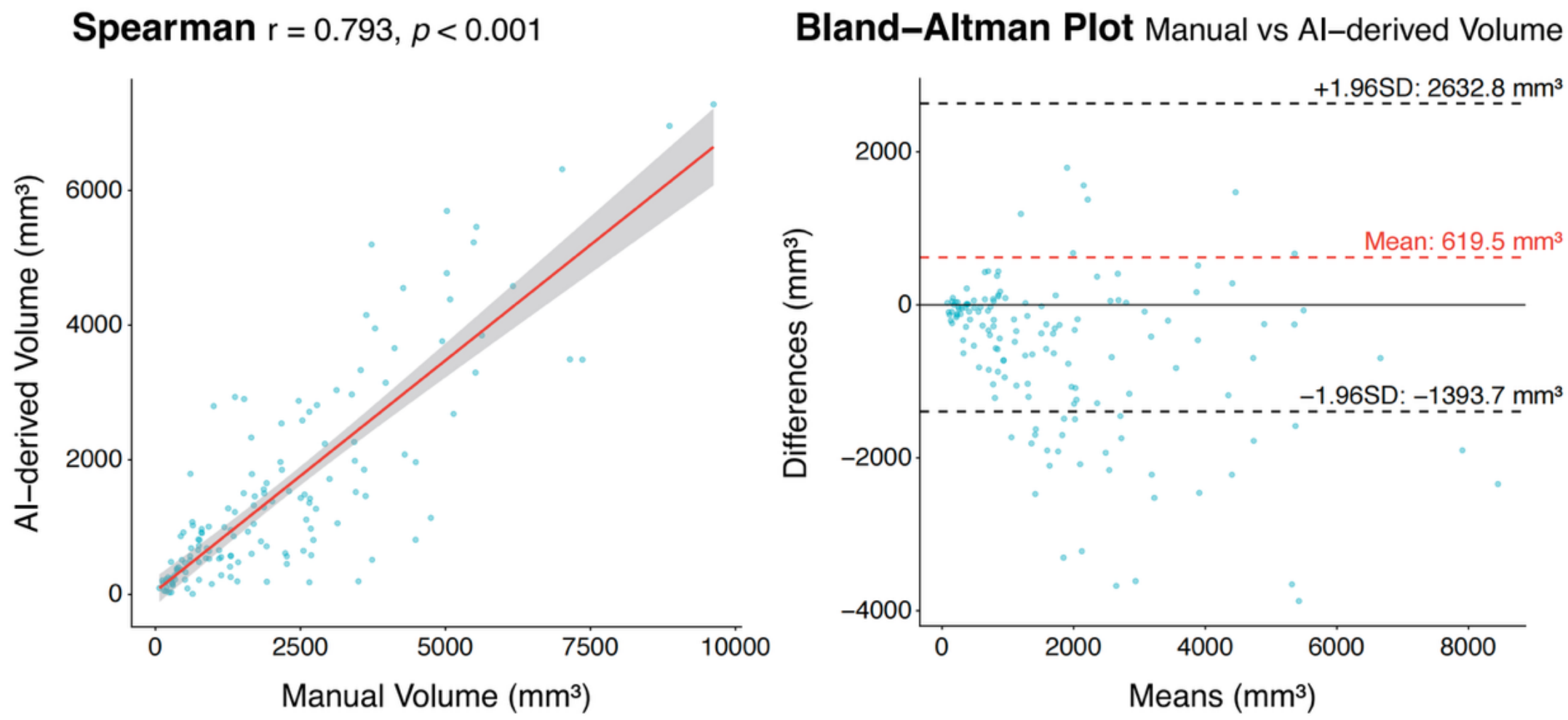

Fig K. Correlation and agreement between AI-derived and manually annotated mrEMVI lesion volumes in true-positive cases. (A) Spearman correlation between AI-derived and manually annotated mrEMVI volumes. The red line represents the linear regression fit with 95% confidence interval (shaded area). **(B)** Bland-Altman plot illustrating the agreement between AI-derived and manually annotated volumes. The red dashed line indicates the mean difference (619.5 mm³), and the black dashed lines indicate the 95% limits of agreement (−1393.7 to 2632.8 mm³). AI, artificial intelligence; mr, magnetic resonance; EMVI, extramural vascular invasion.

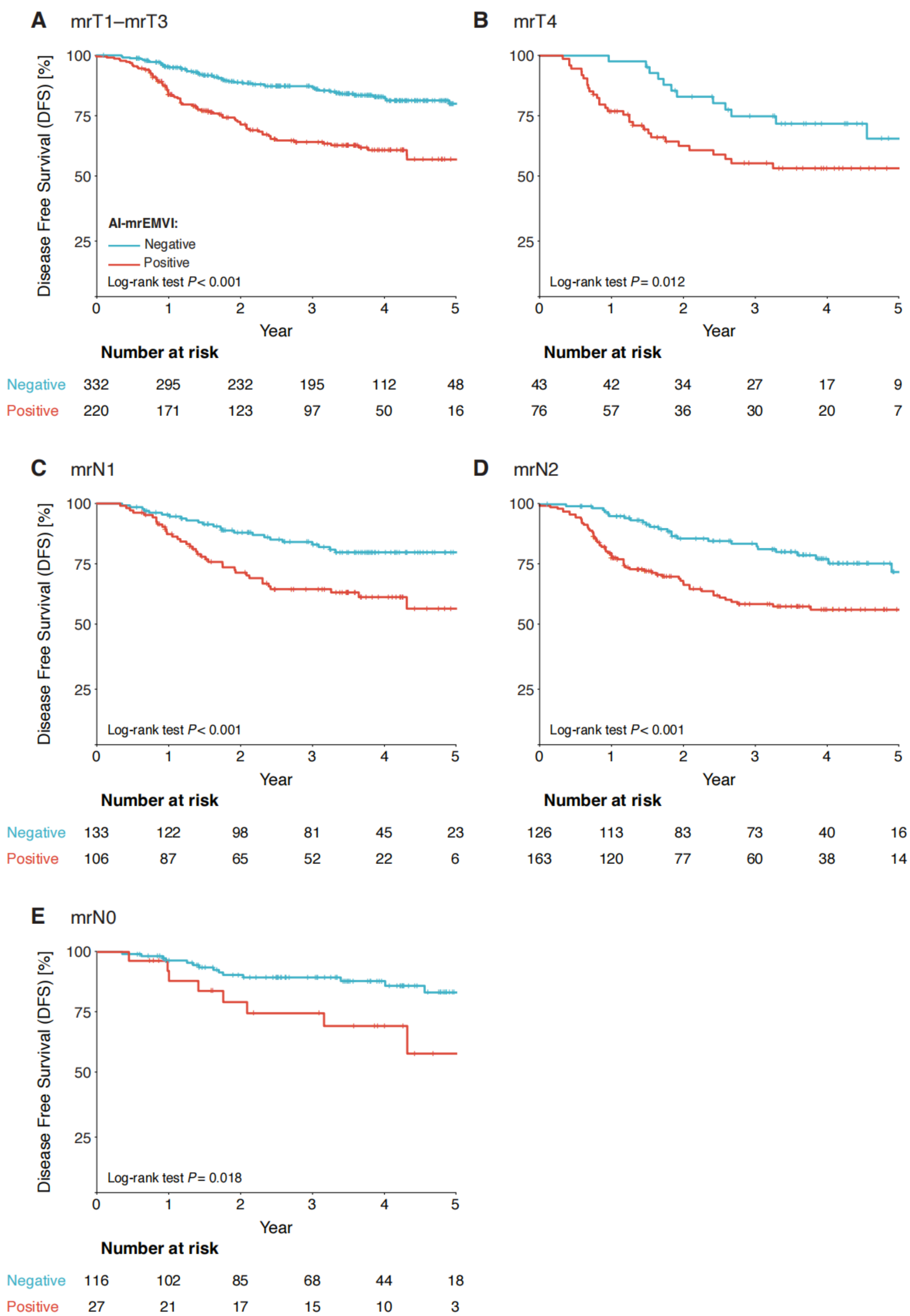

Fig L. Kaplan-Meier curves for DFS according to AI-mrEMVI status in different mrT and mrN staging subgroups. **(A)** mrT1–mrT3. **(B)** mrT4. **(C)** mrN1. **(D)** mrN2. **(E)** mrN0. AI, artificial intelligence; mr, magnetic resonance; EMVI, extramural vascular invasion; DFS, disease-free survival.

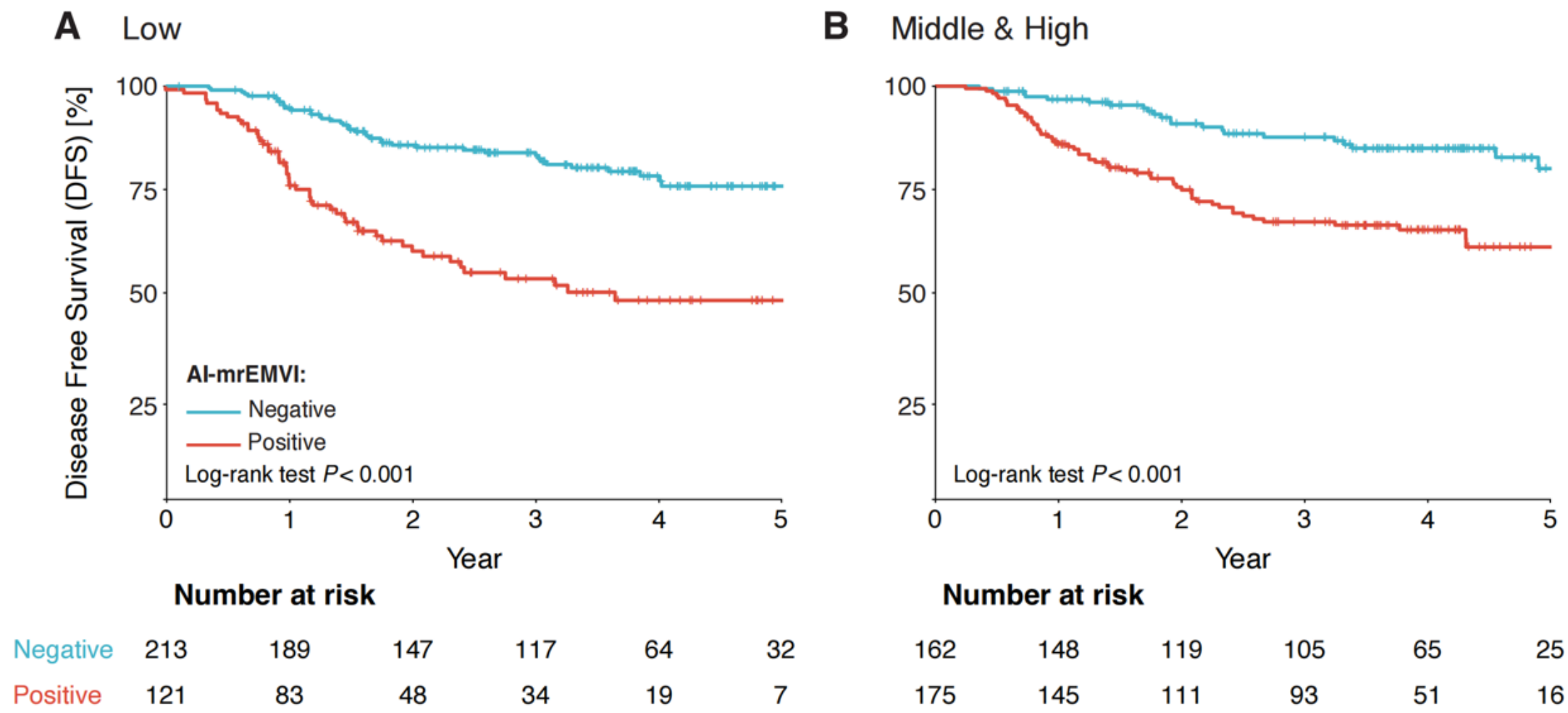

Fig M. Kaplan-Meier curves for DFS according to AI-mrEMVI status in different tumor location subgroups. (A) Low rectal cancer. **(B)** Middle and high rectal cancer. AI, artificial intelligence; mr, magnetic resonance; EMVI, extramural vascular invasion; DFS, disease-free survival.

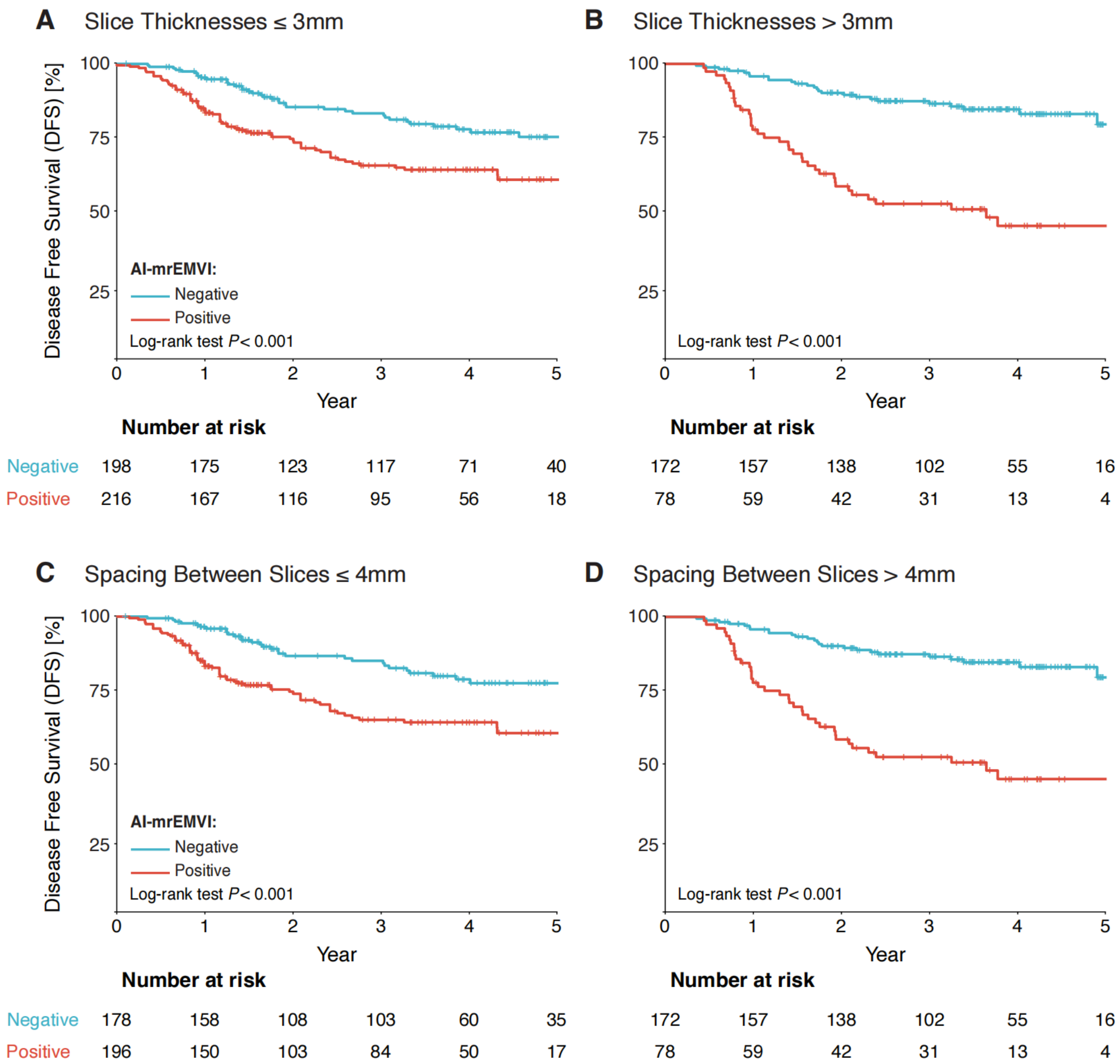

Fig N. Kaplan-Meier curves for DFS according to AI-mrEMVI status in different subgroups of MR T2WI images with varying slice thicknesses and spacing between slices. (A) Slice thicknesses ≤ 3mm. **(B)** Slice thicknesses > 3mm. **(C)** Spacing between slices ≤ 4mm. **(D)** Spacing between slices > 4mm. AI, artificial intelligence; mr, magnetic resonance; EMVI, extramural vascular invasion; DFS, disease-free survival; T2WI, T2-weighted images.

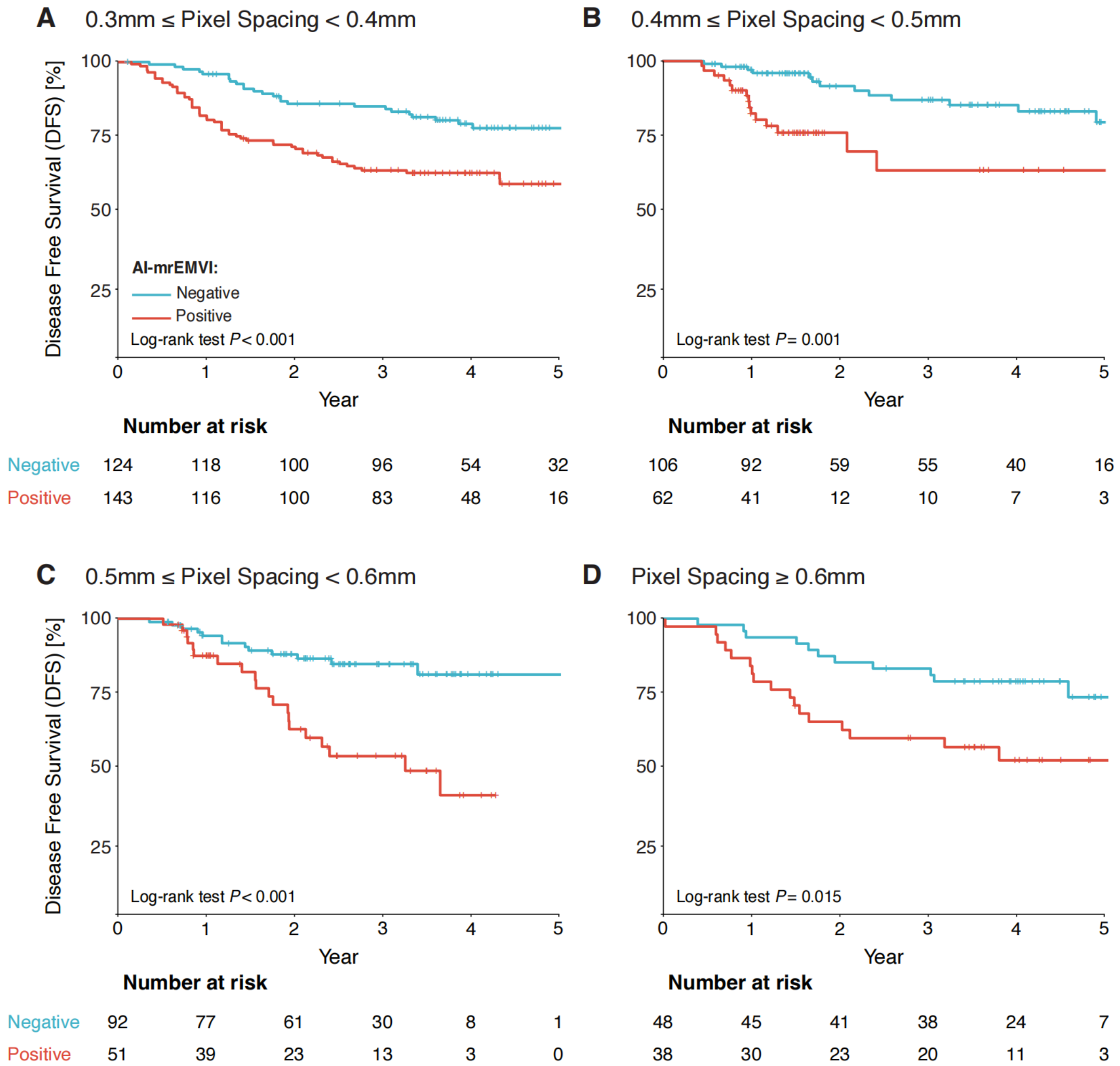

Fig O. Kaplan-Meier curves for DFS according to AI-mrEMVI status in different subgroups of MR T2WI images with varying pixel spacing. (A) 0.3mm ≤ Pixel Spacing < 0.4mm. **(B)** 0.4mm ≤ Pixel Spacing < 0.5mm. **(C)** 0.5mm ≤ Pixel Spacing < 0.6mm. **(D)** Pixel Spacing ≥ 0.6mm. AI, artificial intelligence; mr, magnetic resonance; EMVI, extramural vascular invasion; DFS, disease-free survival; T2WI, T2-weighted images.

Table A. MRI acquisition parameters across the participating centers.

Hospital	Scanner	Sequence	TR/TE[a] (ms)	FOV (mm)	Matrix	Slice Thickness	Slice Gap	Flip Angle
**Guangdong provincial People's Hospital**	Philips 3.0T (Ingenia)	T2WI axial	4300/141.12	100	464×461	4	4	90°
		T2WI sagittal	3000/80	100	300×270	3	3	90°
		T2WI coronal	3128/80	100	316×273	3	3	90°
**The Sixth Affiliated Hospital of Sun Yat-sen University**	GE 3.0T (OPTIMA)	T2WI axial	4300/104	100	288×256	3	6	90°
		T2WI sagittal	3578/104	100	288×256	3	3.5	90°
		T2WI coronal	3782/105.6	100	288×256	3	3.5	90°
**Sun Yat-sen University Cancer Center**	Philips 3.0T (Achieva)	T2WI axial	2852/90	100	516×510	3	4	90°
		T2WI sagittal	3416/90	100	242×139	3	4	90°
		T2WI coronal	2852/100	100	387×222	5	6	90°
	GE 3.0T (DISCOVERY)	T2WI axial	6480/81	100	382×384	4	5	90°
		T2WI sagittal	3883/90	100	436×218	3	4	90°
		T2WI coronal	3916/82	100	512×512	3	4	90°
	GE 1.5T (SIGNA)	T2WI axial	6000/92	60	320×224	5	6	90°
		T2WI sagittal	5903/91	100	512×512	5	6	90°
		T2WI coronal	5916/104	100	343×471	3	4	90°
	SIEMENS 3.0T (Trio Tim)	T2WI axial	3000/84	75	300×230	4	6	90°
		T2WI sagittal	2300/83	100	384×384	3	3.6	90°
		T2WI coronal	4400/81	100	320×320	6	7.2	90°

**Shanxi Cancer Hospital (train cohort)**	Philips 3.0T (Achieva)	T2WI axial	3000/80	100	300×223	3	3.3	90°
		T2WI sagittal	4382.2/70	100	300×267	4	5	90°
		T2WI coronal	3000/80	100	300×218	3	4	90°
**Shanxi Cancer Hospital (external test cohort)**	Philips 3.0T (Achieva)	T2WI axial	3000/80	100	300×223	3	3.3	90°
		T2WI sagittal	4382.2/70	100	300×267	4	5	90°
		T2WI coronal	3000/80	100	300×218	3	4	90°
**the First Affiliated Hospital of Wenzhou Medical University**	Philips 3.0T (Achieva)	T2WI axial	3337/82.8	66	460×448	3	4	90°
		T2WI sagittal	3052/80	70.7	300×296	3	4	90°
		T2WI coronal	3000/80	100	300×285	4	5	90°
**Fudan University Cancer Center**	GE 3.0T (DISCOVERY)	T2WI axial	3920/106	100	512×512	3–4	3.5–5	90°
	SIEMENS 1.5T（Avanto）	T2WI axial	1500/99	100	320×320	1–1.90	3.5–5	90°
**Yunnan Cancer Hospital**	SIEMENS 1.5T（Avanto）	T2WI axial	3200/100	100	288×320	3–4.5	3.3–5.2	90°
	Philips 3.0T (Achieva)	T2WI axial	4000/100	100	432×432	3–5	3.3–6	90°

T2WI, T2-weighted images; TR, Repetition Time; TE, Echo Time; FOV, Field of View.

Table B. Segmentation performance comparison across deep learning architectures in internal five-fold cross-validation.

Model	Tumor Dice (95% CI)	Intravascular Tumor Signal Dice (95% CI)	Dilated Vascular Structure Dice (95% CI)
nnUNet v2	0.850 (0.842–0.858)	0.442 (0.414–0.469)	0.335 (0.316–0.354)
SegResNet	0.368 (0.317–0.419)	0.114 (0.056–0.172)	0.068 (0.039–0.097)
SwinUNETR	0.362 (0.344–0.381)	0.036 (0.024–0.047)	0.052 (0.042–0.062)
UNETR++	0.738 (0.728–0.749)	0.235 (0.226–0.245)	0.196 (0.188–0.203)

Each architecture was configured according to its official recommended settings and default preprocessing pipeline. CI, confidence interval.

Table C. Classification performance comparison across deep learning architectures on the combined external test cohorts.

Model	Accuracy (95% CI)	Sensitivity (95% CI)	Specificity (95% CI)	PPV (95% CI)	NPV (95% CI)	AUC (95% CI)
nnUNet v2	83.3% (80.3%–86.1%)	89.0% (84.3%–92.4%)	80.2% (76.2%–83.7%)	70.9% (65.5%–75.8%)	93.1% (90.0%–95.4%)	0.846 (0.819–0.874)
SegResNet	71.2% (67.7%–74.5%)	69.1% (62.9%–74.6%)	72.4% (68.0%–76.4%)	57.6% (51.8%–63.2%)	81.2% (77.0%–84.8%)	0.707 (0.671–0.744)
SwinUNETR	68.9% (65.2%–72.2%)	43.6% (37.5%–50.0%)	82.5% (78.7%–85.8%)	57.5% (50.2%–64.5%)	73.0% (68.9%–76.7%)	0.631 (0.594–0.667)
UNETR++	51.6% (47.8%–55.3%)	93.2% (89.3%–95.8%)	29.0% (24.9%–33.4%)	41.6% (37.5%–45.8%)	88.7% (82.5%–92.9%)	0.611 (0.584–0.638)

Classification performance was assessed on the combined external test cohorts (n=671). Each architecture was configured according to its official recommended settings. PPV, positive predictive value; NPV, negative predictive value; AUC, area under the receiver operating characteristic curve; CI, confidence interval.

Table D. Delta C-index and 95% confidence intervals for model comparisons in disease-free survival (DFS) and overall survival (OS).

Comparison	Delta C-index	95% CI (DFS)	*P*	Delta C-index	95% CI (OS)	*P*
Clinical model vs Clinical+mrEMVI model	0.0392	0.0123–0.0702	<0.001	0.0352	0.0047–0.0707	0.010
Clinical model vs Clinical+AI-mrEMVI model	0.0572	0.0296–0.0920	<0.001	0.0369	0.0056–0.0730	0.014
Clinical+mrEMVI model vs Clinical+AI-mrEMVI model	0.0180	-0.0045–0.0423	0.138	0.0018	-0.0184–0.0237	0.888

CI, confidence interval; mr, magnetic resonance; EMVI, extramural vascular invasion; AI, artificial intelligence; DFS, disease-free survival; OS, overall survival.

Table E. Univariable and multivariable analyses of OS

	**Overall survival (External Test Cohort 1)**				**Overall survival (External Test Cohort 2)**			
	**Univariable analysis**		**Multivariable analysis**		**Univariable analysis**		**Multivariable analysis**	
	**HR (95% CI)**	***P***	**AHR (95% CI)**	***P***	**HR (95% CI)**	***P***	**AHR (95% CI)**	***P***
Age	1.00 (0.97–1.04)	0.909			1.01 (0.99–1.03)	0.400		
Sex								
Female	Ref				Ref			
Male	1.39 (0.55–3.51)	0.485			0.77 (0.44–1.37)	0.373		
CEA level[a]								
Normal	Ref				Ref			
Abnormal	1.74 (0.78–3.88)	0.178			1.20 (0.72–2.00)	0.496		
Location								
Low	Ref				Ref		Ref	
Middle & High	1.40 (0.62–3.16)	0.415			0.67 (0.42–1.05)	0.082	0.50 (0.31–0.80)	0.004
mrT stage								
T1–T3	Ref				Ref		Ref	
T4	1.59 (0.63–4.01)	0.326			2.61 (1.62–4.20)	< 0.001	2.41 (1.47–3.94)	< 0.001
mrN stage								
N0	Ref				Ref			
N1	2.65 (0.74–9.50)	0.135			1.40 (0.65–3.02)	0.393		
N2	3.20 (0.88–11.66)	0.077			2.16 (1.05–4.45)	0.036		
AI-mrEMVI status								
Negative	Ref		Ref		Ref		Ref	
Positive	3.76 (1.66–8.52)	0.001	3.76 (1.66–8.52)	0.001	2.25 (1.40–3.63)	0.001	2.27 (1.38–3.73)	0.001

HR, hazard ratio; AHR, adjusted hazard ratio; CI, confidence interval; mr, magnetic resonance; CEA, carcinoembryonic antigen; EMVI, extramural vascular invasion; AI, artificial intelligence.

[a]CEA level was categorized as normal (≤5 ng/mL) or abnormal (>5 ng/mL) based on standard clinical thresholds.

Note: *P* value was performed by χ^2 test.

Table F. Schoenfeld residual test results for proportional hazards assumption in multivariable Cox regression models of DFS and OS in two external test cohorts.

Variable	External Test Cohort 1 DFS (chisq, df, *p*)	External Test Cohort 1 OS (chisq, df, *p*)	External Test Cohort 2 DFS (chisq, df, *p*)	External Test Cohort 2 OS (chisq, df, *p*)
AI-mrEMVI	0.344, 1, 0.56	1.290, 1, 0.26	1.633, 1, 0.201	0.108, 1, 0.74
mrT	-	-	-	0.069, 1, 0.79
mrN	-	-	3.431, 1, 0.064	-
Location	-	-	0.094, 2, 0.954	2.857, 2, 0.24
CEA level	1.462, 1, 0.23	-	-	-
GLOBAL	1.612, 2, 0.45	1.290, 1, 0.26	5.022, 4, 0.285	3.114, 4, 0.54

DFS, Disease-free survival; OS, overall survival; AI, artificial intelligence; mr, magnetic resonance; EMVI, extramural vascular invasion; CEA, carcinoembryonic antigen.
